# A consensus prognostic gene expression classifier for ER positive breast cancer

**DOI:** 10.1186/gb-2006-7-10-r101

**Published:** 2006-10-31

**Authors:** Andrew E Teschendorff, Ali Naderi, Nuno L Barbosa-Morais, Sarah E Pinder, Ian O Ellis, Sam Aparicio, James D Brenton, Carlos Caldas

**Affiliations:** 1 Cancer Genomics Program, Department of Oncology, University of Cambridge, Hutchison/MRC Research Center, Hills Road, Cambridge CB2 2XZ, UK; 2 Institute of Molecular Medicine, Faculty of Medicine, University of Lisbon, 1649-028 Lisbon, Portugal; 3 Cancer Genomics Program, Department of Pathology, University of Cambridge, Hutchison/MRC Research Center, Hills Road, Cambridge CB2 2XZ, UK; 4 Histopathology, Nottingham City Hospital NHS Trust and University of Nottingham, Nottingham NG5 1PB, UK; 5 Molecular Oncology and Breast Cancer Program, the BC Cancer Research Centre, West 10th Avenue, Vancouver BC, V5Z 1L3, Canada

## Abstract

**Background:**

A consensus prognostic gene expression classifier is still elusive in heterogeneous diseases such as breast cancer.

**Results:**

Here we perform a combined analysis of three major breast cancer microarray data sets to hone in on a universally valid prognostic molecular classifier in estrogen receptor (ER) positive tumors. Using a recently developed robust measure of prognostic separation, we further validate the prognostic classifier in three external independent cohorts, confirming the validity of our molecular classifier in a total of 877 ER positive samples. Furthermore, we find that molecular classifiers may not outperform classical prognostic indices but that they can be used in hybrid molecular-pathological classification schemes to improve prognostic separation.

**Conclusion:**

The prognostic molecular classifier presented here is the first to be valid in over 877 ER positive breast cancer samples and across three different microarray platforms. Larger multi-institutional studies will be needed to fully determine the added prognostic value of molecular classifiers when combined with standard prognostic factors.

## Abstract 2

A consensus prognostic classifier for estrogen receptor positive breast tumors has been developed and shown to be valid in nearly 900 samples across different microarray platforms.

## Background

The identification of a prognostic gene expression signature in breast cancer that is valid across multiple independent data sets and different microarray platforms is a challenging problem [1]. Recently, there have been reports of molecular prognostic and predictive signatures that were also valid in external independent cohorts [2-7]. One of these studies derived the prognostic signature from genes correlating with histological grade [4], while in [5] it was derived directly from correlations with clinical outcome data and was validated in estrogen receptor positive lymph node negative (ER+LN-) breast cancer. Another study validated a predictive score, based on 21 genes, for ER+LN-tamoxifen treated breast cancer [2]. These results are encouraging, yet, as explained recently in [8,9], much larger cohort sizes may be needed before a consensus prognostic signature emerges. While the intrinsic subtype classification does appear to constitute a set of consensus signatures [7], it is also clear that these classifiers are not optimized for prognosis. Moreover, although different prognostic signatures have recently been shown to give similar classifications in one breast cancer cohort [6], this result was not shown to hold in other cohorts. In fact, a problem remains in that the two main prognostic gene signatures derived so far [10,11] do not validate in the other's data set, even when cohort differences are taken into account [9,12]. Furthermore, the 21 genes that make up the predictive score [2] were derived from a relatively small number of genes (approximately 250) using criteria such as assay-probe performance. Hence, it is likely that other gene combinations could result in improved classifiers. These problems have raised questions about the clinical utility of molecular signatures as currently developed [13].

There are many factors that may contribute to the observed lack of consistency between derived signatures. In addition to cohort size, another factor is the use of dichotomized outcome variables, a procedure that is justified clinically but which may introduce significant bias [14]. A related problem concerns the way molecular prognostic classifiers have been evaluated, which is often done by dichotomizing the associated molecular prognostic index (MPI). Such dichotomizations are often not justified since they implicitly assume a bi-modal distribution for the MPI, while the evidence points at prognostic indices that are often best described in terms of uni-modal distributions [4,10,11]. Another difficulty concerns the evaluation of a prognostic index in external independent studies, which requires a careful recalibration procedure, but which is often either ignored or not addressed rigorously [15]. A strategy that may allow for uni-modal prognostic index distributions and that allows a more objective and reliable evaluation of a prognostic classifier across independent cohorts is, therefore, desirable [16].

Another matter of recent controversy is whether a molecular prognostic signature can outperform classical prognostic factors, such as lymph node status, tumor size, grade or combinations thereof such as the Nottingham Prognostic Index (NPI) [17]. It was shown that molecular prognostic signatures are the strongest predictors in multivariate Cox-regression models that include standard prognostic factors [4,5,18,19]. On the other hand, more objective tests that compare a molecular prognostic signature with classical prognostic factors in completely independent cohorts profiled on different platforms is still lacking. Furthermore, it appears that prognostic models that combine classical prognostic factors in multivariate models may perform as well, or even better than, molecular prognostic signatures [20].

One way to effectively increase the cohort size is to use a combined ('meta-analysis') approach. Meta-analyses of microarray data sets have already enabled identification of robust metagene signatures associated with neoplastic transformation and progression and particular gene functions across a wide range of different tumor types [21,22]. A meta-analysis of breast cancer was also recently attempted [23], where four independent breast cancer cohorts were fused together using an ingenious Bayesian method [24], and from which a metasignature was derived that correlated with relapse in each of the four studies. This study was exploratory in nature, however, and did not evaluate the metasignature in independent data sets. Furthermore, the metasignature was derived from a mix of ER+ and ER-tumors and was, therefore, confounded by ER status. In fact, this signature does not validate in the more recent breast cancer cohorts (Teschendorff AE, unpublished).

In this work we present a combined analysis of ER+ breast cancer that uses a recently proposed framework [16] for objectively evaluating prognostic separation of a molecular classifier across independent data sets and platforms. Importantly, this evaluation method does not dichotomize the prognostic index, allowing for prognostic index distributions that may be uni-modal. Using this novel approach, the purpose of our work is two-fold. First, to hone in on a consensus set of prognostic genes by using a meta-analysis to derive a prognostic molecular classifier in ER+ breast cancer and show that it validates in completely independent external cohorts and different platforms. Second, to evaluate its prognostic separation relative to histopathological prognostic factors and to explore the prognostic added value of molecular classifiers when combined with classical prognostic factors. We use six of the largest breast cancer cohorts available (described in [4,11,12,18,25,26]; in [4] we used the independent cohort of 101 samples from the John Radcliffe Hospital, Oxford, UK), representing a total of 877 ER+ patients profiled across three different microarray platforms.

## Results

**Figure 1:**
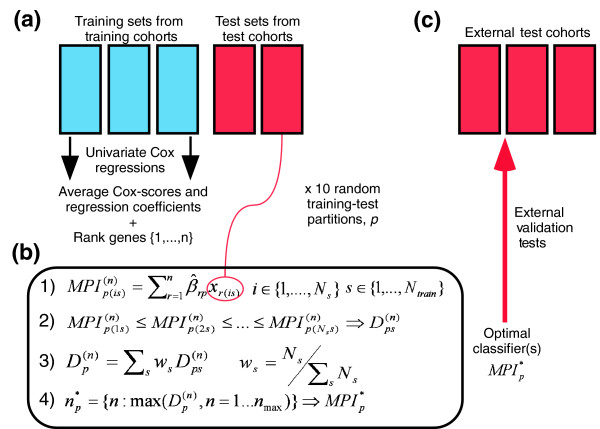
**(a) **For each of 10 random partitions of training cohorts into training and test sets we rank the genes according to their average Cox-scores over the *N*_*train *_training cohorts (*N*_*train *_= 3). **(b) **1, Definition of MPI and evaluation of the optimal classifier(s) using the independent test sets of the training cohorts. 2, Dps(n)
 MathType@MTEF@5@5@+=feaafiart1ev1aaatCvAUfeBSjuyZL2yd9gzLbvyNv2Caerbhv2BYDwAHbqedmvETj2BSbqee0evGueE0jxyaibaiKI8=vI8tuQ8FMI8Gi=hEeeu0xXdbba9frFj0=OqFfea0dXdd9vqai=hGuQ8kuc9pgc9s8qqaq=dirpe0xb9q8qiLsFr0=vr0=vr0dc8meaabaqaciGacaGaaeqabaqadeqadaaakeaacaWGebWaa0baaSqaaiaadchacaWGZbaabaGaaiikaiaad6gacaGGPaaaaaaa@385C@ denotes the D-index of the top *n*-gene classifier for partition/realization *p *in test set of the training cohort *s*. 3, Dp(n)
 MathType@MTEF@5@5@+=feaafiart1ev1aaatCvAUfeBSjuyZL2yd9gzLbvyNv2Caerbhv2BYDwAHbqedmvETj2BSbqee0evGueE0jxyaibaiKI8=vI8tuQ8FMI8Gi=hEeeu0xXdbba9frFj0=OqFfea0dXdd9vqai=hGuQ8kuc9pgc9s8qqaq=dirpe0xb9q8qiLsFr0=vr0=vr0dc8meaabaqaciGacaGaaeqabaqadeqadaaakeaacaWGebWaa0baaSqaaiaadchaaeaacaGGOaGaamOBaiaacMcaaaaaaa@3764@ denotes the weighted average D-index over the test sets in the training cohorts where *N*_*s *_denotes the size of the test set of training cohort *s*. 4, The optimal classifier for each partition/realization *p*, MPIp∗
 MathType@MTEF@5@5@+=feaafiart1ev1aaatCvAUfeBSjuyZL2yd9gzLbvyNv2Caerbhv2BYDwAHbqedmvETj2BSbqee0evGueE0jxyaibaiKI8=vI8tuQ8FMI8Gi=hEeeu0xXdbba9frFj0=OqFfea0dXdd9vqai=hGuQ8kuc9pgc9s8qqaq=dirpe0xb9q8qiLsFr0=vr0=vr0dc8meaabaqaciGacaGaaeqabaqadeqadaaakeaacaWGnbGaamiuaiaadMeadaqhaaWcbaGaamiCaaqaaiabgEHiQaaaaaa@37B3@, is defined by the number of top-ranked genes, *n*, that maximizes Dp(n)
 MathType@MTEF@5@5@+=feaafiart1ev1aaatCvAUfeBSjuyZL2yd9gzLbvyNv2Caerbhv2BYDwAHbqedmvETj2BSbqee0evGueE0jxyaibaiKI8=vI8tuQ8FMI8Gi=hEeeu0xXdbba9frFj0=OqFfea0dXdd9vqai=hGuQ8kuc9pgc9s8qqaq=dirpe0xb9q8qiLsFr0=vr0=vr0dc8meaabaqaciGacaGaaeqabaqadeqadaaakeaacaWGebWaa0baaSqaaiaadchaaeaacaGGOaGaamOBaiaacMcaaaaaaa@3764@. **(c) **Validation of the optimal classifiers MPIp∗
 MathType@MTEF@5@5@+=feaafiart1ev1aaatCvAUfeBSjuyZL2yd9gzLbvyNv2Caerbhv2BYDwAHbqedmvETj2BSbqee0evGueE0jxyaibaiKI8=vI8tuQ8FMI8Gi=hEeeu0xXdbba9frFj0=OqFfea0dXdd9vqai=hGuQ8kuc9pgc9s8qqaq=dirpe0xb9q8qiLsFr0=vr0=vr0dc8meaabaqaciGacaGaaeqabaqadeqadaaakeaacaWGnbGaamiuaiaadMeadaqhaaWcbaGaamiCaaqaaiabgEHiQaaaaaa@37B3@ in completely independent external cohorts.

The six microarray data sets used are summarized in Table 1 by platform type, number of ER+ samples and outcome events. Following the recommendations set out in [1], we did not use all data sets to train a molecular classifier but left some out to provide us with completely independent test sets. Our overall strategy is summarized in Figure 1. We decided to use as training cohorts the two largest available cohorts (NKI2 and EMC) [11,18] in addition to our own data set (NCH) [12], amounting to 527 ER+ samples (with 146 poor outcome events) profiled over 5,007 common genes. This choice was motivated by our previous work [12], where a prognostic signature, derived from the NCH cohort, was found to be prognostic in the NKI2 cohort and marginally prognostic in the ECM cohort, suggesting that, by combining the three cohorts (NKI2, ECM and NCH) in a meta-analysis, an improved classifier could be potentially derived. As external test sets we used the three cohorts JRH-1 [25], JRH-2 [4] and UPP [26], giving a total of 350 ER+ test samples (with 86 poor outcome events). Time to overall survival was used as outcome endpoint, except for the two cohorts EMC and JRH-2, where this clinical information was unavailable and time to distant metastasis (TTDM) was used instead.

### A meta-analysis derived molecular prognostic index (MPI)

The derivation of the molecular classifier is described in detail in Materials and methods (see also Figure 1). Briefly, each of the three training cohorts was divided into 10 different training-test set partitions [27], ensuring the same number of training samples for each training cohort. Because of the small cohort size of NCH (*n *= 93), all samples from this cohort were used; thus, 93 training samples were also used from the NKI2 and EMC cohorts. We found that, by choosing a smaller training set for NCH, the performance of the classifier in the NCH test set would be too variable and would unduly influence the derived prognostic classifier. While using the whole NCH cohort as a training set introduces a slight bias towards selecting features that perform well in the NCH cohort, this is offset by optimizing the classifier to the test sets in NKI2 and EMC. The remaining samples in NKI2 (*n *= 133) and EMC (*n *= 115) were used as additional independent test sets. The common genes were z-score normalized and ranked, for each training-test set partition *p *= 1,...,10, according to their average univariate Cox-scores over the three training data sets. A continuous molecular prognostic index (MPI_*p*_) for each of the test samples (*i*) in the training cohorts (*s*) and for a given number of top-ranked genes in the classifier (*n*) was then computed by the dot product of the average Cox-regression coefficient vector (β^
 MathType@MTEF@5@5@+=feaafiart1ev1aaatCvAUfeBSjuyZL2yd9gzLbvyNv2Caerbhv2BYDwAHbqedmvETj2BSbqee0evGueE0jxyaibaiKI8=vI8tuQ8FMI8Gi=hEeeu0xXdbba9frFj0=OqFfea0dXdd9vqai=hGuQ8kuc9pgc9s8qqaq=dirpe0xb9q8qiLsFr0=vr0=vr0dc8meaabaqaciGacaGaaeqabaqadeqadaaakeaaiiGacuWFYoGygaqcaaaa@34E5@_*gp*_, (*g *= 1,..., *n*)) (as estimated from the training-set samples) with the vector of normalized gene expression values (*x*_*gis*_, (*g *= 1,..., *n*)), that is:

MPIisp=∑g=1nβ^gpxgis
MathType@MTEF@5@5@+=feaafiart1ev1aaatCvAUfeBSjuyZL2yd9gzLbvyNv2Caerbhv2BYDwAHbqedmvETj2BSbqee0evGueE0jxyaibaiKI8=vI8tuQ8FMI8Gi=hEeeu0xXdbba9frFj0=OqFfea0dXdd9vqai=hGuQ8kuc9pgc9s8qqaq=dirpe0xb9q8qiLsFr0=vr0=vr0dc8meaabaqaciGacaGaaeqabaqadeqadaaakeaaieaacaWFnbGaa8huaiaa=LeadaWgaaWcbaGaamyAaiaadohacaWGWbaabeaakiabg2da9maapehabaGafqOSdiMbaKaadaWgaaWcbaGaam4zaiaadchaaeqaaOGaamiEamaaBaaaleaacaWGNbGaamyAaiaadohaaeqaaaqaaiaadEgacqGH9aqpcaaIXaaabaGaamOBaaqdcqGHris5aaaa@4750@

This is explained in more detail in Materials and methods. Prognostic separation of the classifiers was then evaluated using a novel robust measure, the D-index, as recently proposed [16]. The D-index, which depends only on the relative risk ordering of the test samples as determined by their continuous MPI values, can be interpreted as a robust generalized hazard ratio [16]. A weighted average D-index (the weights were chosen proportional to the number of test-samples in each cohort) over the two test sets in NKI2 and EMC was then computed and its variation as a function of the number of top-ranked genes in the classifier is shown in Additional data file 1 for two different training-test set partitions. For each of the ten partitions, an optimal number of genes (39, 99, 63, 53, 43, 84, 70, 27, 33, 18) could be readily identified, and the performance of the optimal classifiers in the two test sets was highly significant (range of weighted average D-index was 2.25 to 3.32 and all log-rank test p values < 0.05; see also Table 2). The fact that the genes, ranked using the training sets, formed classifiers that were prognostic in the independent test sets and that this result was stable under changes in the composition of the training-test sets used indicated to us that a universally valid prognostic classifier could be potentially derived [27].

### A consensus molecular prognostic classifier

**Figure 2:**
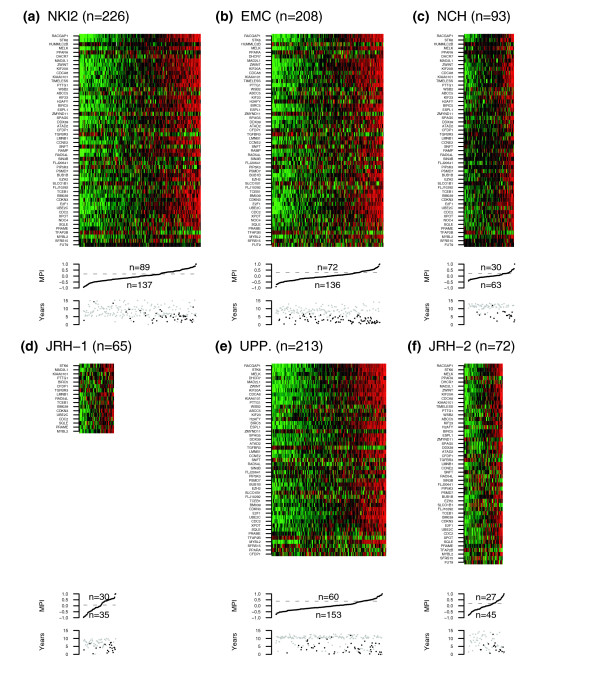
The MPI in the training and test cohorts. Heatmaps of relative gene expression (green = 'low', red = 'high') of the optimal 52-gene classifier and accompanying MPI distribution values across the three training cohorts **(a) **NKI2, **(b) **EMC and **(c) **NCH, and three test cohorts **(d) **JRH-1, **(e) **UPP and **(f) **JRH-2. The threshold shown for the MPI distributions was determined as explained in the text. Lower panels show the survival time distributions in the respective cohorts (black = 'death/poor outcome', grey = 'censored/good outcome').

To arrive at a final list of prognostic genes, independent of any choice of training-test set realization, we computed the global average Cox-scores over the ten training-test set realizations and three training cohorts. The resulting global averaged Cox-scores were then used to give a final ranking of the genes. A 'consensus' optimal classifier was then built by sequentially adding genes from the top of this list to a classifier set and computing the D-index of this classifier for each of the three training cohorts. An overall D-index score, *D*_*O*_, was then evaluated as the weighted average of the D-indices for each training cohort (*D*_*S*_), that is:

Do=∑s∈StrainwsDs
 MathType@MTEF@5@5@+=feaafiart1ev1aaatCvAUfeBSjuyZL2yd9gzLbvyNv2Caerbhv2BYDwAHbqedmvETj2BSbqee0evGueE0jxyaibaiKI8=vI8tuQ8FMI8Gi=hEeeu0xXdbba9frFj0=OqFfea0dXdd9vqai=hGuQ8kuc9pgc9s8qqaq=dirpe0xb9q8qiLsFr0=vr0=vr0dc8meaabaqaciGacaGaaeqabaqadeqadaaakeaacaWGebWaaSbaaSqaaiaad+gaaeqaaOGaeyypa0ZaaabeaeaacaWG3bWaaSbaaSqaaiaadohaaeqaaOGaamiramaaBaaaleaacaWGZbaabeaaaeaacaWGZbGaeyicI4Saam4uamaaBaaameaacaWG0bGaamOCaiaadggacaWGPbGaamOBaaqabaaaleqaniabggHiLdaaaa@4458@

where the weights are in direct proportion to the number of samples in each cohort. The overall D-index value, as a function of the number of top-ranked genes, is shown in Additional data file 2. This identified an 'optimal' classifier of 52 genes (Table 2; Figure 2a-c; Additional data file 3) with an overall D-index value of 3.71 (95% confidence interval (CI) 2.16 to 6.58; p < 10^-6^). It is noteworthy that the classifier based on the top 17 genes (Table 3) achieved similar prognostic performance (Table 2; Additional data file 2), with an overall D-index value of 3.70.

### Validation in three external cohorts

We next validated the 17-gene and 52-gene classifiers in the three external independent cohorts JRH-1, UPP and JRH2. The MPI associated with these classifiers induced in each of these cohorts an ordering based on the relative risks of the samples. As before, the association of the predicted risk ordering with outcome was tested by computing the D-indices and the corresponding log-rank test p values yielded their levels of significance. Remarkably, both classifiers were valid in the three external independent cohorts JRH-1, UPP and JRH-2 and performed equally well (Table 2), with statistically significant D-index values (for the 52-gene classifier) of 3.44 (95%CI 1.67 to 7.00; p < 10^-3^), 2.80 (95%CI 1.73 to 4.54; p < 10^-4^) and 11.26 (95%CI 3.66 to 34.57; p < 10^-5^), respectively. The distribution of MPI values in these cohorts as well as heatmaps of gene expression of our optimal classifier confirmed the robustness of the classifier across different cohorts and platforms (Figure 2d-f). To further test the robustness of this result, we also evaluated the 10 optimal classifiers (Cp∗
 MathType@MTEF@5@5@+=feaafiart1ev1aaatCvAUfeBSjuyZL2yd9gzLbvyNv2Caerbhv2BYDwAHbqedmvETj2BSbqee0evGueE0jxyaibaiKI8=vI8tuQ8FMI8Gi=hEeeu0xXdbba9frFj0=OqFfea0dXdd9vqai=hGuQ8kuc9pgc9s8qqaq=dirpe0xb9q8qiLsFr0=vr0=vr0dc8meaabaqaciGacaGaaeqabaqadeqadaaakeaacaWGdbWaa0baaSqaaiaadchaaeaacqGHxiIkaaaaaa@3606@, *p *= 1,..., 10) in the three external cohorts JRH-1, UPP and JRH-2. The median D-index and the median p value over the 10 Cp∗
 MathType@MTEF@5@5@+=feaafiart1ev1aaatCvAUfeBSjuyZL2yd9gzLbvyNv2Caerbhv2BYDwAHbqedmvETj2BSbqee0evGueE0jxyaibaiKI8=vI8tuQ8FMI8Gi=hEeeu0xXdbba9frFj0=OqFfea0dXdd9vqai=hGuQ8kuc9pgc9s8qqaq=dirpe0xb9q8qiLsFr0=vr0=vr0dc8meaabaqaciGacaGaaeqabaqadeqadaaakeaacaWGdbWaa0baaSqaaiaadchaaeaacqGHxiIkaaaaaa@3606@ classifiers in each of these cohorts are shown in Table 2, which also provides a comparison with the D-indices for the standard prognostic factors in ER+ breast cancer. Over all 10 Cp∗
 MathType@MTEF@5@5@+=feaafiart1ev1aaatCvAUfeBSjuyZL2yd9gzLbvyNv2Caerbhv2BYDwAHbqedmvETj2BSbqee0evGueE0jxyaibaiKI8=vI8tuQ8FMI8Gi=hEeeu0xXdbba9frFj0=OqFfea0dXdd9vqai=hGuQ8kuc9pgc9s8qqaq=dirpe0xb9q8qiLsFr0=vr0=vr0dc8meaabaqaciGacaGaaeqabaqadeqadaaakeaacaWGdbWaa0baaSqaaiaadchaaeaacqGHxiIkaaaaaa@3606@ classifiers, the D-index ranged from 2.04 to 3.96 in JRH-1, from 2.39 to 3.04 in UPP, and from 5.08 to 12.61 in JRH-2, with p values in all cases statistically significant (p < 0.05). It is noteworthy that all 10 molecular classifiers Cp∗
 MathType@MTEF@5@5@+=feaafiart1ev1aaatCvAUfeBSjuyZL2yd9gzLbvyNv2Caerbhv2BYDwAHbqedmvETj2BSbqee0evGueE0jxyaibaiKI8=vI8tuQ8FMI8Gi=hEeeu0xXdbba9frFj0=OqFfea0dXdd9vqai=hGuQ8kuc9pgc9s8qqaq=dirpe0xb9q8qiLsFr0=vr0=vr0dc8meaabaqaciGacaGaaeqabaqadeqadaaakeaacaWGdbWaa0baaSqaaiaadchaaeaacqGHxiIkaaaaaa@3606@ predicted prognosis in the external sets as well as in the independent test sets of the training cohorts (Table 2), a strong indication that the molecular classifiers were not overfitted to the training data.

**Figure 3:**
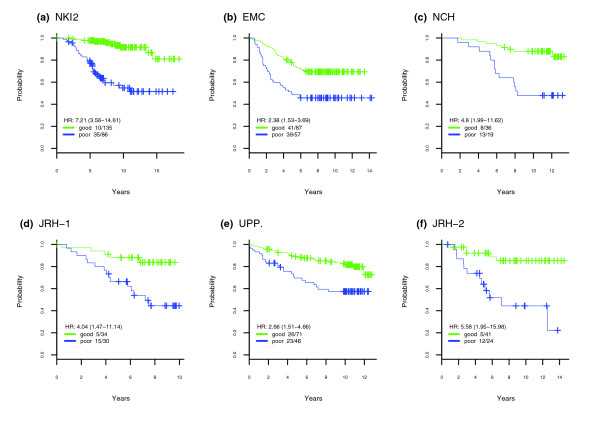
Kaplan-Meier survival curves in cohorts. Kaplan-Meier survival curves for the two prognostic groups derived from pam-clustering (k = 2) [28] on the molecular prognostic index distribution in the three training cohorts **(a) **NKI2, **(b) **EMC and **(c) **NCH, and three external cohorts **(d) **JRH-1, **(e) **UPP and **(f) **JRH-2. We also give the hazard ratio (HR), the associated 95%CI and the number of events (death or distant metastasis) and number of distinct data points in each prognostic group.

In order to relate the D-index scores to well-known performance measures, such as the hazard ratio and survival rates, the MPI profiles need to be dichotomized. Because the D-index framework does not use a cut-off, the dichotomization cannot be done prospectively. Instead, cut-offs can be found for each data set by applying an unsupervised clustering algorithm to the MPI profiles. Specifically, here we applied the partitioning around medoids algorithm (pam) [28] with two centers to learn two prognostic groups in each of the cohorts. Thus, the cut-offs obtained are cohort-dependent but are not necessarily optimized for prognostic performance, as we verified explicitly (data not shown). The resulting Kaplan-Meier survival curves and associated hazard ratios confirmed the significantly different prognostic risks of the two groups (Figure 3). Thus, the MPI identified in each of the external cohorts a low-risk subgroup with a survival rate at 10 years of over 80%, and a high-risk subgroup with a corresponding 10 year survival rate of less than 50%, with the exception of Uppsala's cohort, where the high risk subgroup was less well defined, with a 10 year survival rate of approximately 60%.

### Molecular versus classical prognostic indices

Table 2 also shows that the molecular prognostic classification did not outperform standard histopathological prognostic factors. Notably, in two of the external studies it did not outperform a modified NPI [17] (see Materials and methods), which was overall the best prognostic indicator.

To test whether the molecular prognostic classifiers performed independently of these other histopathological factors, we computed the D-indices in the multivariate Cox setting. In four out of ten realizations the MPI was a significant prognostic predictor (p < 0.05) in JRH-1, in nine out of ten realizations it was significant in UPP, while in JRH-2 it was significant in all realizations (Table 4). Similarly, the optimal 52-gene classifier remained significant in multivariate analysis in two of the external cohorts (UPP and JRH-2), while it failed only marginally in JRH-1 (Table 4). Interestingly, the MPI was the most consistent prognostic predictor across studies.

### Hybrid models to evaluate prognostic added value of MPI

Given that the optimal molecular prognostic classifier derived from over 527 ER+ samples did not outperform histopathological prognostic factors, we next asked whether it could improve prognostic separation in hybrid models in which the standard pathological indices (SPIs) are augmented by the MPI. With a continuous index, such as the NPI or tumor size, a natural way to augment the SPI within the D-index framework is to rank the external samples based on a weighted average ranking over the predicted SPI and MPI rankings (see Materials and methods). We found that, in almost all equal-weight hybrid prognostic models, there was an improvement in prognostic separation when the MPI was added to the SPI (Table 5; Additional data files 4 and 5). However, it is noteworthy that, with the exception of JRH-2, where only 36 samples with NPI information were available, there was no marked improvement when the MPI was added to the NPI, which is consistent with the stronger prognostic performance of the NPI. For the variable-weight models there were only two cases (JRH-1 node status and JRH-2 size) in which a non-hybrid classifier performed best, and in both cases it was the MPI (Additional data file 6). Thus, it appears that, while the MPI added prognostic value to single pathological factors, there was no significant improvement when added to the NPI.

### Gene Ontology

Enrichment of gene ontologies among the top 100 prognostic genes was studied using the Gene Ontology (GO) Tree Machine (GOTM) [29]. Not surprisingly, and in agreement with previous studies [11,18], most of the genes (23/100, p < 10^-9^) were associated with mitotic cell-cycle functions. In terms of molecular function, nucleid acid and ATP binding was also significantly overrepresented (26/100, p < 10^-3^). Furthermore, most genes were associated with intracellular component (62/100, p < 10^-4^). Interestingly, other significantly overrepresented biological processes included microtubule cytoskeleton organization and biogenesis and DNA metabolism. Similar results were obtained for the top 150 and 200 prognostic genes. Summary gene functions for the top 17 and 52 prognostic genes are shown in Table 3 and Additional data file 3, respectively, while the detailed summaries can be found in Additional data files 7, 8, 9.

### Overlap with other prognostic gene lists

Finally, we considered the overlap of our 52 prognostic classifier with the four main molecular prognostic gene lists presented in [4,10-12] (Additional data file 10). Interestingly, the strongest overlap was with the 97 gene list reported in [4], where we found 20 genes in common, and which may explain the better prognostic performance in this cohort, although a mere sample size effect cannot be excluded. Among these 20 genes are well-known prognostic genes in breast cancer (for example, *BIRC5*, *BUB1B*, *CDC2*, *MAD2L1*, *MYBL2*, *STK6*). The overlap with the other three prognostic signatures was weaker: a 2-gene overlap (*ATAD2*, *CCNE2*) with the 76-gene signature of [11], an 8-gene overlap (*CCNE2*, *BIRC5*, *STK6*, *EZH2*, *BM039*, *PSMD7*, *PRAME*, *MAD2L1*) with the 231 prognostic genes of [10], and a 12-gene overlap with the 70-gene signature of [12].

## Discussion

The D-index [15,16] has three key properties that make it particularly suited as a measure of prognostic separation. First, it does not require the MPI to be recalibrated since it is invariant under monotonic transformations that preserve the risk-ordering of samples. Second, because it does not require the MPI to be dichotomized, it allows for uni-modal MPI distributions. Indeed, using various pattern recognition algorithms [30,31], we verified that bi-modality is very often absent from the MPI profiles. Third, because it doesn't use a prospectively defined cut-off it avoids the pitfalls associated with using such a cut-off when evaluating the prognostic performance of a classifier in external cohorts of widely different characteristics. Thus, the D-index provides a more reliable and objective measure of prognostic separation for evaluating classifiers across multiple independent data sets and platforms than, for example, the hazard ratio or the area under the curve. While dichotomization of a prognostic index into good and poor prognostic classes is necessary for clinical decision making, for the purposes of our work dichotomization of the MPI was not necessary.

Using the D-index in a meta-analysis of three ER+ breast cancer microarray data sets, we derived an optimal molecular classifier of 52 genes with an associated rule for computing a MPI and successfully validated it in three completely independent external cohorts. Moreover, we showed that a slightly less optimal but much simpler classifier made up of only 17 genes performed comparably to the 52-gene classifier across all six studies.

The optimal 52-gene classifier showed a notable overlap of 20 genes with the grade-derived prognostic signature reported in [4], which is perhaps not surprising given that the latter signature was prognostic in up to 5 breast cancer cohorts. Intriguingly though, the grade-derived signature was not validated in a large available cohort [11], raising doubts as to its wider applicability. Importantly, and in spite of the significant overlap between our optimal classifier and the grade-derived signature reported in [4], we found that our optimal classifier performed independently of grade. In addition, we verified that our optimal classifier performed independently of the ER gene expression level (data not shown) in ER+ tumors. The overlap of the 52-gene classifier with either van't Veer's or Wang's prognostic signature was smaller, yet these two signatures also fail to validate in each other's data set. We believe that all these results strongly support the validity of the 52-gene and 17-gene prognostic signatures and that we have successfully honed in on a core set of prognostic genes for ER+ breast cancer, to be tested further in prospective clinical studies.

The D-index also provided us with a framework in which to objectively evaluate the molecular prognostic index against classical prognostic factors in external cohorts. We found that molecular classifiers may increase prognostic separability when added to single prognostic factors, such as grade or node status. However, in agreement with [20], we didn't find the molecular prognostic index to either outperform or add prognostic value to the NPI. In fact, our analyses showed that the degree of improvement in prognostic separation over the NPI was strongly dependent on the cohort considered, indicating that larger cohorts of more uniform characteristics will be needed to rigorously elucidate the future clinical role of molecular prognostic classifiers in breast cancer.

## Conclusion

The molecular classifier derived here is the first molecular prognostic classification scheme that is valid across six major breast cancer studies representing a total of 877 ER+ patients profiled over three different platforms. In order to further test this prognostic classifier and to fully evaluate the prognostic value it adds over standard prognostic factors such as the NPI, we propose a multi-institutional study that profiles the consensus set of genes identified here over larger and more homogeneous cohorts using either quantitative RT-PCR or custom-made arrays.

## Materials and methods

### Internal data set

The cohort of 135 primary breast tumors was profiled using Agilent Human 1A 60-mer (Agilent Technologies, Santa Clara, CA, USA) oligonucleotide microarrays containing 22,575 features (19,061 genes and 3,514 control spots) [12]. Details regarding RNA amplification, labeling, hybridization and scanning are as described previously [32,33]. Feature extraction, normalization of the raw data and data filtering were performed using the Agilent G2567AA Feature Extraction software (Agilent Technologies) and Spotfire DecisionSite 8.0 (Somerville, MA, USA). This resulted in a normalized matrix of 8,278 genes (Additional data file 11). The clinical data are also summarized in Additional data file 11.

### External data sets and gene annotation

The external microarray breast cancer data sets considered in this work are described in [4,11,18,25,26]. For these cohorts we used the normalized data, which are available in the public domain (see references). The retrieved data sets were further normalized, if necessary, by transforming them onto a common log2-scale and shifting the median of each array to zero. We also created an automated computational pipeline (Perl scripts on a Linux platform) to cross-link the annotation provided for each dataset with UniGene. For some datasets, the linkage relied on Ensembl [34] external database identifiers. Thus, each probe was associated with a universal gene name. This procedure generated a non-redundant set of gene identifiers for the subsequent meta-analysis.

### The D-index measure for prognostic separation

Here, we briefly review the D-index measure for prognostic separation as proposed in [16]. A classifier *C *induces on a set of *n *samples with gene expression vectors (x→1,x→2,...,x→n
 MathType@MTEF@5@5@+=feaafiart1ev1aaatCvAUfeBSjuyZL2yd9gzLbvyNv2Caerbhv2BYDwAHbqedmvETj2BSbqee0evGueE0jxyaibaiKI8=vI8tuQ8FMI8Gi=hEeeu0xXdbba9frFj0=OqFfea0dXdd9vqai=hGuQ8kuc9pgc9s8qqaq=dirpe0xb9q8qiLsFr0=vr0=vr0dc8meaabaqaciGacaGaaeqabaqadeqadaaakeaaceWG4bGbaSaadaWgaaWcbaGaaGymaaqabaGccaGGSaGabmiEayaalaWaaSbaaSqaaiaaikdaaeqaaOGaaiilaiaac6cacaGGUaGaaiOlaiaacYcaceWG4bGbaSaadaWgaaWcbaGaamOBaaqabaaaaa@3D82@) a 'risk ordering' based on the relative magnitude of the continuous prognostic indices *PI*_*k *_= *PI*(x→κ
 MathType@MTEF@5@5@+=feaafiart1ev1aaatCvAUfeBSjuyZL2yd9gzLbvyNv2Caerbhv2BYDwAHbqedmvETj2BSbqee0evGueE0jxyaibaiKI8=vI8tuQ8FMI8Gi=hEeeu0xXdbba9frFj0=OqFfea0dXdd9vqai=hGuQ8kuc9pgc9s8qqaq=dirpe0xb9q8qiLsFr0=vr0=vr0dc8meaabaqaciGacaGaaeqabaqadeqadaaakeaaceWG4bGbaSaadaWgaaWcbaGaeqOUdSgabeaaaaa@361A@) (*k *= 1,..., *n*). Given outcome data *O *= (*T *× *E*)^*n*^, where *T *∈ [0, *t*_*max*_] and *E *∈ {0,1} represent the time to event and event type random variables, respectively, one may evaluate the prognostic separation predicted by *C *by a Cox-proportional hazards regression:

*log*(*h*_*i*_(*t*)) = *log*(*h*_0_(*t*)) + *PI*_*i *_∀*i *= 1,..., *n*.     (2)

and estimating the log-rank test p value. A difficulty with this approach is that, generally, the prognostic index needs recalibrating in the independent data sets where prognostic separation is to be evaluated. To overcome this difficulty a robust measure of prognostic separation that does not need model recalibration has been proposed. It is obtained by considering only the relative risk ordering of the samples and then evaluating this risk ordering against the actual outcome data. Specifically, let us assume that *C *induces the ordering (i_1_, i_2_,..., i_*n*_), so that PIi1≤PIi2≤...≤PIin
 MathType@MTEF@5@5@+=feaafiart1ev1aaatCvAUfeBSjuyZL2yd9gzLbvyNv2Caerbhv2BYDwAHbqedmvETj2BSbqee0evGueE0jxyaibaiKI8=vI8tuQ8FMI8Gi=hEeeu0xXdbba9frFj0=OqFfea0dXdd9vqai=hGuQ8kuc9pgc9s8qqaq=dirpe0xb9q8qiLsFr0=vr0=vr0dc8meaabaqaciGacaGaaeqabaqadeqadaaakeaacaWGqbGaamysamaaBaaaleaacaWGPbWaaSbaaWqaaiaaigdaaeqaaaWcbeaakiabgsMiJkaadcfacaWGjbWaaSbaaSqaaiaadMgadaWgaaadbaGaaGOmaaqabaaaleqaaOGaeyizImQaaiOlaiaac6cacaGGUaGaeyizImQaamiuaiaadMeadaWgaaWcbaGaamyAamaaBaaameaacaWGUbaabeaaaSqabaaaaa@45BF@. Assume further that the *PI*_*i *_are normally distributed (this assumption is not crucial to the argument as similar results hold for PI that are not normally distributed [16]), so that they can be expressed in terms of the standard gaussian (ordered) rankits (*u*_1_,..., *u*_*n*_) as:

PIij
 MathType@MTEF@5@5@+=feaafiart1ev1aaatCvAUfeBSjuyZL2yd9gzLbvyNv2Caerbhv2BYDwAHbqedmvETj2BSbqee0evGueE0jxyaibaiKI8=vI8tuQ8FMI8Gi=hEeeu0xXdbba9frFj0=OqFfea0dXdd9vqai=hGuQ8kuc9pgc9s8qqaq=dirpe0xb9q8qiLsFr0=vr0=vr0dc8meaabaqaciGacaGaaeqabaqadeqadaaakeaacaWGqbGaamysamaaBaaaleaacaWGPbWaaSbaaWqaaiaadQgaaeqaaaWcbeaaaaa@3711@ = μ + σ*u*_*j *_+ ε_*j *_    (3)

where ε_*j *_denote the error terms, μ is the mean of the PI distribution and σ denotes the standard deviation of the PI and is a direct measure of prognostic separation. A robust measure of prognostic separation can now be obtained by regressing the outcome data against the scaled rankits:

zj=π8uj
 MathType@MTEF@5@5@+=feaafiart1ev1aaatCvAUfeBSjuyZL2yd9gzLbvyNv2Caerbhv2BYDwAHbqedmvETj2BSbqee0evGueE0jxyaibaiKI8=vI8tuQ8FMI8Gi=hEeeu0xXdbba9frFj0=OqFfea0dXdd9vqai=hGuQ8kuc9pgc9s8qqaq=dirpe0xb9q8qiLsFr0=vr0=vr0dc8meaabaqaciGacaGaaeqabaqadeqadaaakeaacaWG6bWaaSbaaSqaaiaadQgaaeqaaOGaeyypa0ZaaOqaaeaadaWcaaqaaiabec8aWbqaaiaaiIdaaaaaleqaaOGaamyDamaaBaaaleaacaWGQbaabeaaaaa@3B21@

that is,

*log*(hij
 MathType@MTEF@5@5@+=feaafiart1ev1aaatCvAUfeBSjuyZL2yd9gzLbvyNv2Caerbhv2BYDwAHbqedmvETj2BSbqee0evGueE0jxyaibaiKI8=vI8tuQ8FMI8Gi=hEeeu0xXdbba9frFj0=OqFfea0dXdd9vqai=hGuQ8kuc9pgc9s8qqaq=dirpe0xb9q8qiLsFr0=vr0=vr0dc8meaabaqaciGacaGaaeqabaqadeqadaaakeaacaWGObWaaSbaaSqaaiaadMgadaWgaaadbaGaamOAaaqabaaaleqaaaaa@365B@(*t*)) = *b*(*t*) + σ*z_*j *_∀*j *= 1,..., *n*.     (4)

and estimating the coefficient σ*. Note that the mean μ has been absorbed into the baseline hazard function. As explained in more detail in [16], the scaling of the rankits ensures the interpretability of σ* as a generalized log-hazard ratio. We adopt here a slightly different convention to [16] and define the D-index, *D*, as *D *≡ eσ*
 MathType@MTEF@5@5@+=feaafiart1ev1aaatCvAUfeBSjuyZL2yd9gzLbvyNv2Caerbhv2BYDwAHbqedmvETj2BSbqee0evGueE0jxyaibaiKI8=vI8tuQ8FMI8Gi=hEeeu0xXdbba9frFj0=OqFfea0dXdd9vqai=hGuQ8kuc9pgc9s8qqaq=dirpe0xb9q8qiLsFr0=vr0=vr0dc8meaabaqaciGacaGaaeqabaqadeqadaaakeaacaWGLbWaaWbaaSqabeaacqaHdpWCdaahaaadbeqaaiaacQcaaaaaaaaa@36E3@.

The D-index, in contrast to the hazard ratio (HR) [35] and the Brier Score [36], combines interpretability, precision (confidence intervals can be readily computed) and robustness (because it only depends on the relative risk ordering of the samples, it is invariant under monotonic recalibration transformations). Ties in the PI are treated by averaging the corresponding rankits as explained in [16]. In the extreme case of a binary prognostic index *PI *∈ {0,1}, it can be shown that *D *≤ *HR *and *D *≈ *HR *when the imbalance between 1s and 0s is small.

### Derivation of the molecular prognostic index

We first decided which data sets to use for training and deriving an optimal molecular prognostic classifier and which to leave out for external independent validation tests. Denoting *S*_*train *_and *S*_*test *_as the set of training studies and test studies, respectively, we then divided each of the cohorts in *S*_*train *_randomly into 10 different training-test set partitions. The partitioning was performed ensuring equal proportions of events (death or distant metastasis) in training and test sets and to ensure approximately equal numbers of training samples in each training cohort. Next, genes were normalized to have mean zero and unit standard deviation across the training samples in each training cohort separately. For each training cohort and training set we then performed univariate Cox-regressions over the *G *genes common to all studies in *S*_*train*_. Let β→
 MathType@MTEF@5@5@+=feaafiart1ev1aaatCvAUfeBSjuyZL2yd9gzLbvyNv2Caerbhv2BYDwAHbqedmvETj2BSbqee0evGueE0jxyaibaiKI8=vI8tuQ8FMI8Gi=hEeeu0xXdbba9frFj0=OqFfea0dXdd9vqai=hGuQ8kuc9pgc9s8qqaq=dirpe0xb9q8qiLsFr0=vr0=vr0dc8meaabaqaciGacaGaaeqabaqadeqadaaakeaacuaHYoGygaWcaaaa@34E0@_*sp *_and Q→
 MathType@MTEF@5@5@+=feaafiart1ev1aaatCvAUfeBSjuyZL2yd9gzLbvyNv2Caerbhv2BYDwAHbqedmvETj2BSbqee0evGueE0jxyaibaiKI8=vI8tuQ8FMI8Gi=hEeeu0xXdbba9frFj0=OqFfea0dXdd9vqai=hGuQ8kuc9pgc9s8qqaq=dirpe0xb9q8qiLsFr0=vr0=vr0dc8meaabaqaciGacaGaaeqabaqadeqadaaakeaaceWGrbGbaSaaaaa@3415@_*sp *_denote, for study *s *∈ *S*_*train *_and partition *p *∈ {1,...,10}, the vectors of *G *estimated regression coefficients and *G *Cox-scores, respectively. We then computed for each partition *p *the average coefficient vector β^→
 MathType@MTEF@5@5@+=feaafiart1ev1aaatCvAUfeBSjuyZL2yd9gzLbvyNv2Caerbhv2BYDwAHbqedmvETj2BSbqee0evGueE0jxyaibaiKI8=vI8tuQ8FMI8Gi=hEeeu0xXdbba9frFj0=OqFfea0dXdd9vqai=hGuQ8kuc9pgc9s8qqaq=dirpe0xb9q8qiLsFr0=vr0=vr0dc8meaabaqaciGacaGaaeqabaqadeqadaaakeaacuaHYoGygaqcgaWcaaaa@34EF@_*p *_and average Cox-score vector Q^→
 MathType@MTEF@5@5@+=feaafiart1ev1aaatCvAUfeBSjuyZL2yd9gzLbvyNv2Caerbhv2BYDwAHbqedmvETj2BSbqee0evGueE0jxyaibaiKI8=vI8tuQ8FMI8Gi=hEeeu0xXdbba9frFj0=OqFfea0dXdd9vqai=hGuQ8kuc9pgc9s8qqaq=dirpe0xb9q8qiLsFr0=vr0=vr0dc8meaabaqaciGacaGaaeqabaqadeqadaaakeaaceWGrbGbaKGbaSaaaaa@3424@ as:

β^→p≡1|Strain|∑s∈Strainβ→sp     (5)
MathType@MTEF@5@5@+=feaafiart1ev1aaatCvAUfeBSjuyZL2yd9gzLbvyNv2Caerbhv2BYDwAHbqedmvETj2BSbqee0evGueE0jxyaibaiKI8=vI8tuQ8FMI8Gi=hEeeu0xXdbba9frFj0=OqFfea0dXdd9vqai=hGuQ8kuc9pgc9s8qqaq=dirpe0xb9q8qiLsFr0=vr0=vr0dc8meaabaqaciGacaGaaeqabaqadeqadaaakeaacuaHYoGygaqcgaWcamaaBaaaleaacaWGWbaabeaakiabggMi6oaalaaabaGaaGymaaqaaiaacYhacaWGtbWaaSbaaSqaaiaadshacaWGYbGaamyyaiaadMgacaWGUbaabeaakiaacYhaaaWaa8qCaeaacuaHYoGygaWcamaaBaaaleaacaWGZbGaamiCaaqabaaabaGaam4CaiabgIGiolaadofadaWgaaadbaGaamiDaiaadkhacaWGHbGaamyAaiaad6gaaeqaaaWcbeqdcqGHris5aOGaaCzcaiaaxMaadaqadaqaaiaaiwdaaiaawIcacaGLPaaaaaa@5250@

and similarly for Q^→
 MathType@MTEF@5@5@+=feaafiart1ev1aaatCvAUfeBSjuyZL2yd9gzLbvyNv2Caerbhv2BYDwAHbqedmvETj2BSbqee0evGueE0jxyaibaiKI8=vI8tuQ8FMI8Gi=hEeeu0xXdbba9frFj0=OqFfea0dXdd9vqai=hGuQ8kuc9pgc9s8qqaq=dirpe0xb9q8qiLsFr0=vr0=vr0dc8meaabaqaciGacaGaaeqabaqadeqadaaakeaaceWGrbGbaKGbaSaaaaa@3424@_*p*_. We next ranked for each partition *p *the *G *genes according to their average Cox-scores across the training studies. Let r→
 MathType@MTEF@5@5@+=feaafiart1ev1aaatCvAUfeBSjuyZL2yd9gzLbvyNv2Caerbhv2BYDwAHbqedmvETj2BSbqee0evGueE0jxyaibaiKI8=vI8tuQ8FMI8Gi=hEeeu0xXdbba9frFj0=OqFfea0dXdd9vqai=hGuQ8kuc9pgc9s8qqaq=dirpe0xb9q8qiLsFr0=vr0=vr0dc8meaabaqaciGacaGaaeqabaqadeqadaaakeaaceWGYbGbaSaaaaa@3436@_*p *_= (*r*_1*p*_,..., *r*_*Gp*_) where *r*_*gp *_specifies, for partition *p*, the position in {1,..., *G*} of the *g*th ranked gene. The following procedure was then carried out for each partition *p *to obtain an optimal molecular classifier Cp∗
 MathType@MTEF@5@5@+=feaafiart1ev1aaatCvAUfeBSjuyZL2yd9gzLbvyNv2Caerbhv2BYDwAHbqedmvETj2BSbqee0evGueE0jxyaibaiKI8=vI8tuQ8FMI8Gi=hEeeu0xXdbba9frFj0=OqFfea0dXdd9vqai=hGuQ8kuc9pgc9s8qqaq=dirpe0xb9q8qiLsFr0=vr0=vr0dc8meaabaqaciGacaGaaeqabaqadeqadaaakeaacaWGdbWaa0baaSqaaiaadchaaeaacqGHxiIkaaaaaa@3606@:

First, let *n *denote the number of top ranked genes in the classifier set. Set *n *= 1.

Second, for every test sample *i *in each of the training cohorts *s *we compute a molecular prognostic index:

MPIisp(n)=∑g=1nβ^rgppxrgpis
 MathType@MTEF@5@5@+=feaafiart1ev1aaatCvAUfeBSjuyZL2yd9gzLbvyNv2Caerbhv2BYDwAHbqedmvETj2BSbqee0evGueE0jxyaibaiKI8=vI8tuQ8FMI8Gi=hEeeu0xXdbba9frFj0=OqFfea0dXdd9vqai=hGuQ8kuc9pgc9s8qqaq=dirpe0xb9q8qiLsFr0=vr0=vr0dc8meaabaqaciGacaGaaeqabaqadeqadaaakeaaieaacaWFnbGaa8huaiaa=LeadaqhaaWcbaGaamyAaiaadohacaWGWbaabaGaaiikaiaad6gacaGGPaaaaOGaeyypa0ZaaabmaeaacuaHYoGygaqcamaaBaaaleaacaWGYbWaaSbaaWqaaiaadEgacaWGWbaabeaaliaadchaaeqaaOGaamiEamaaBaaaleaacaWGYbWaaSbaaWqaaiaadEgacaWGWbaabeaaliaadMgacaWGZbaabeaaaeaacaWGNbGaeyypa0JaaGymaaqaaiaad6gaa0GaeyyeIuoaaaa@4DA5@

where xrgpis
 MathType@MTEF@5@5@+=feaafiart1ev1aaatCvAUfeBSjuyZL2yd9gzLbvyNv2Caerbhv2BYDwAHbqedmvETj2BSbqee0evGueE0jxyaibaiKI8=vI8tuQ8FMI8Gi=hEeeu0xXdbba9frFj0=OqFfea0dXdd9vqai=hGuQ8kuc9pgc9s8qqaq=dirpe0xb9q8qiLsFr0=vr0=vr0dc8meaabaqaciGacaGaaeqabaqadeqadaaakeaacaWG4bWaaSbaaSqaaiaadkhadaWgaaadbaGaam4zaiaadchaaeqaaSGaamyAaiaadohaaeqaaaaa@394C@ denotes the normalized expression of gene *r*_*gp *_in sample *i *of training study *s *∈ *S*_*train*_. The expression normalization is done using the mean and standard deviation from the training set.

Third, for each partition *p *and training study *s *we compute the D-index on the test samples as explained in the previous subsection. This yields a value Dsp(n)
 MathType@MTEF@5@5@+=feaafiart1ev1aaatCvAUfeBSjuyZL2yd9gzLbvyNv2Caerbhv2BYDwAHbqedmvETj2BSbqee0evGueE0jxyaibaiKI8=vI8tuQ8FMI8Gi=hEeeu0xXdbba9frFj0=OqFfea0dXdd9vqai=hGuQ8kuc9pgc9s8qqaq=dirpe0xb9q8qiLsFr0=vr0=vr0dc8meaabaqaciGacaGaaeqabaqadeqadaaakeaacaWGebWaa0baaSqaaiaadohacaWGWbaabaGaaiikaiaad6gacaGGPaaaaaaa@385C@.

Fourth, compute the average D-index over the training studies:

D^p(n)=∑s∈StrainwsDsp(n)
 MathType@MTEF@5@5@+=feaafiart1ev1aaatCvAUfeBSjuyZL2yd9gzLbvyNv2Caerbhv2BYDwAHbqedmvETj2BSbqee0evGueE0jxyaibaiKI8=vI8tuQ8FMI8Gi=hEeeu0xXdbba9frFj0=OqFfea0dXdd9vqai=hGuQ8kuc9pgc9s8qqaq=dirpe0xb9q8qiLsFr0=vr0=vr0dc8meaabaqaciGacaGaaeqabaqadeqadaaakeaaceWGebGbaKaadaqhaaWcbaGaamiCaaqaaiaacIcacaWGUbGaaiykaaaakiabg2da9maaqababaGaam4DamaaBaaaleaacaWGZbaabeaakiaadseadaqhaaWcbaGaam4CaiaadchaaeaacaGGOaGaamOBaiaacMcaaaaabaGaam4CaiabgIGiolaadofadaWgaaadbaGaamiDaiaadkhacaWGHbGaamyAaiaad6gaaeqaaaWcbeqdcqGHris5aaaa@49F8@

where *w*_*s *_denotes the weights for each training study to take account of the varying numbers of samples in the test sets of the training studies.

Fifth, increment *n *by 1 and repeat steps two to five until *n *≤ *n*_*max*_.

Sixth, let np∗
 MathType@MTEF@5@5@+=feaafiart1ev1aaatCvAUfeBSjuyZL2yd9gzLbvyNv2Caerbhv2BYDwAHbqedmvETj2BSbqee0evGueE0jxyaibaiKI8=vI8tuQ8FMI8Gi=hEeeu0xXdbba9frFj0=OqFfea0dXdd9vqai=hGuQ8kuc9pgc9s8qqaq=dirpe0xb9q8qiLsFr0=vr0=vr0dc8meaabaqaciGacaGaaeqabaqadeqadaaakeaacaWGUbWaa0baaSqaaiaadchaaeaacqGHxiIkaaaaaa@3631@ = {*n *: *max*(D^p(n)
 MathType@MTEF@5@5@+=feaafiart1ev1aaatCvAUfeBSjuyZL2yd9gzLbvyNv2Caerbhv2BYDwAHbqedmvETj2BSbqee0evGueE0jxyaibaiKI8=vI8tuQ8FMI8Gi=hEeeu0xXdbba9frFj0=OqFfea0dXdd9vqai=hGuQ8kuc9pgc9s8qqaq=dirpe0xb9q8qiLsFr0=vr0=vr0dc8meaabaqaciGacaGaaeqabaqadeqadaaakeaaceWGebGbaKaadaqhaaWcbaGaamiCaaqaaiaacIcacaWGUbGaaiykaaaaaaa@3774@) *and n *≥ 10} denote the optimal number of top-ranked genes. Thus, for each partition *p *we have an optimal classifier Cp∗
 MathType@MTEF@5@5@+=feaafiart1ev1aaatCvAUfeBSjuyZL2yd9gzLbvyNv2Caerbhv2BYDwAHbqedmvETj2BSbqee0evGueE0jxyaibaiKI8=vI8tuQ8FMI8Gi=hEeeu0xXdbba9frFj0=OqFfea0dXdd9vqai=hGuQ8kuc9pgc9s8qqaq=dirpe0xb9q8qiLsFr0=vr0=vr0dc8meaabaqaciGacaGaaeqabaqadeqadaaakeaacaWGdbWaa0baaSqaaiaadchaaeaacqGHxiIkaaaaaa@3606@ defined by a set of np∗
 MathType@MTEF@5@5@+=feaafiart1ev1aaatCvAUfeBSjuyZL2yd9gzLbvyNv2Caerbhv2BYDwAHbqedmvETj2BSbqee0evGueE0jxyaibaiKI8=vI8tuQ8FMI8Gi=hEeeu0xXdbba9frFj0=OqFfea0dXdd9vqai=hGuQ8kuc9pgc9s8qqaq=dirpe0xb9q8qiLsFr0=vr0=vr0dc8meaabaqaciGacaGaaeqabaqadeqadaaakeaacaWGUbWaa0baaSqaaiaadchaaeaacqGHxiIkaaaaaa@3631@ genes and associated average regression coefficients {β^r1pp,...,β^rnp*pp
 MathType@MTEF@5@5@+=feaafiart1ev1aaatCvAUfeBSjuyZL2yd9gzLbvyNv2Caerbhv2BYDwAHbqedmvETj2BSbqee0evGueE0jxyaibaiKI8=vI8tuQ8FMI8Gi=hEeeu0xXdbba9frFj0=OqFfea0dXdd9vqai=hGuQ8kuc9pgc9s8qqaq=dirpe0xb9q8qiLsFr0=vr0=vr0dc8meaabaqaciGacaGaaeqabaqadeqadaaakeaacuaHYoGygaqcamaaBaaaleaacaWGYbWaaSbaaWqaaiaaigdacaWGWbaabeaaliaadchaaeqaaOGaaiilaiaac6cacaGGUaGaaiOlaiaacYcacuaHYoGygaqcamaaBaaaleaacaWGYbWaaSbaaWqaaiaad6gadaqhaaqaaiaadchaaeaacaGGQaaaaiaadchaaeqaaSGaamiCaaqabaaaaa@440C@} and the rule in step-2 for computing a continuous MPI for independent samples.

Two notes with this procedure are in order: *n*_*max *_can be estimated by evaluating the statistical significance of the average ranking position of the common genes across the training studies - for our purposes, setting *n*_*max *_= 100 led to suitable optimal classifiers; and we constrained n* to be larger than 10 in order to ensure a significant number of overlapping genes for the independent validation tests.

### Validation in independent data sets

The above procedure yields for each choice of training-test set partition in the training studies a molecular classifier and an associated rule for computing a continuous molecular prognostic index for independent test samples. For an independent test sample *i *in test study *s *∈ *S*_*test *_we compute its molecular prognostic index as:

MPIisp*=∑g=1np*β^rgppxrgpis     (6)
MathType@MTEF@5@5@+=feaafiart1ev1aaatCvAUfeBSjuyZL2yd9gzLbvyNv2Caerbhv2BYDwAHbqedmvETj2BSbqee0evGueE0jxyaibaiKI8=vI8tuQ8FMI8Gi=hEeeu0xXdbba9frFj0=OqFfea0dXdd9vqai=hGuQ8kuc9pgc9s8qqaq=dirpe0xb9q8qiLsFr0=vr0=vr0dc8meaabaqaciGacaGaaeqabaqadeqadaaakeaaieaacaWFnbGaa8huaiaa=LeadaqhaaWcbaGaamyAaiaadohacaWGWbaabaGaaiOkaaaakiabg2da9maapehabaGafqOSdiMbaKaadaWgaaWcbaGaamOCamaaBaaameaacaWGNbGaamiCaaqabaWccaWGWbaabeaakiaadIhadaWgaaWcbaGaamOCamaaBaaameaacaWGNbGaamiCaaqabaWccaWGPbGaam4CaaqabaaabaGaam4zaiabg2da9iaaigdaaeaacaWGUbWaa0baaWqaaiaadchaaeaacaGGQaaaaaqdcqGHris5aOGaaCzcaiaaxMaadaqadaqaaiaaiAdaaiaawIcacaGLPaaaaaa@51AF@

where xrgpis
 MathType@MTEF@5@5@+=feaafiart1ev1aaatCvAUfeBSjuyZL2yd9gzLbvyNv2Caerbhv2BYDwAHbqedmvETj2BSbqee0evGueE0jxyaibaiKI8=vI8tuQ8FMI8Gi=hEeeu0xXdbba9frFj0=OqFfea0dXdd9vqai=hGuQ8kuc9pgc9s8qqaq=dirpe0xb9q8qiLsFr0=vr0=vr0dc8meaabaqaciGacaGaaeqabaqadeqadaaakeaacaWG4bWaaSbaaSqaaiaadkhadaWgaaadbaGaam4zaiaadchaaeqaaSGaamyAaiaadohaaeqaaaaa@394C@ has been normalized as before. Recalibration of the MPI values is necessary if a prognostic meaning is to be attached to these values. This is difficult because of inter-cohort variability. The D-index measure of prognostic separation, however, overcomes this difficulty since it only depends on the relative risk ordering of the external samples. Thus, for each test study *s *and partition *p *we can evaluate the prognostic separation of the molecular classifier Cp∗
 MathType@MTEF@5@5@+=feaafiart1ev1aaatCvAUfeBSjuyZL2yd9gzLbvyNv2Caerbhv2BYDwAHbqedmvETj2BSbqee0evGueE0jxyaibaiKI8=vI8tuQ8FMI8Gi=hEeeu0xXdbba9frFj0=OqFfea0dXdd9vqai=hGuQ8kuc9pgc9s8qqaq=dirpe0xb9q8qiLsFr0=vr0=vr0dc8meaabaqaciGacaGaaeqabaqadeqadaaakeaacaWGdbWaa0baaSqaaiaadchaaeaacqGHxiIkaaaaaa@3606@ by computing the D-index *D*_*sp *_and the associated log-rank test p values.

### Hybrid or augmented prognostic models

To evaluate whether the molecular prognostic index improved prognostic separation over histopathological classifiers we considered hybrid augmented models within the D-index framework. Specifically, we assume that we have a rule to compute a MPI (as given in the last section) for a sample *i *in a new cohort, which we denote by *MPI*_*i*_. Suppose further we have a histopathological prognostic index value for each sample *i *in this new cohort, which we denote by *SPI*_*i*_. Both indices induce a relative risk ordering of the samples. Let riM
 MathType@MTEF@5@5@+=feaafiart1ev1aaatCvAUfeBSjuyZL2yd9gzLbvyNv2Caerbhv2BYDwAHbqedmvETj2BSbqee0evGueE0jxyaibaiKI8=vI8tuQ8FMI8Gi=hEeeu0xXdbba9frFj0=OqFfea0dXdd9vqai=hGuQ8kuc9pgc9s8qqaq=dirpe0xb9q8qiLsFr0=vr0=vr0dc8meaabaqaciGacaGaaeqabaqadeqadaaakeaacaWGYbWaa0baaSqaaiaadMgaaeaacaWGnbaaaaaa@3611@ and riP
 MathType@MTEF@5@5@+=feaafiart1ev1aaatCvAUfeBSjuyZL2yd9gzLbvyNv2Caerbhv2BYDwAHbqedmvETj2BSbqee0evGueE0jxyaibaiKI8=vI8tuQ8FMI8Gi=hEeeu0xXdbba9frFj0=OqFfea0dXdd9vqai=hGuQ8kuc9pgc9s8qqaq=dirpe0xb9q8qiLsFr0=vr0=vr0dc8meaabaqaciGacaGaaeqabaqadeqadaaakeaacaWGYbWaa0baaSqaaiaadMgaaeaacaWGqbaaaaaa@3614@ denote the rank position of sample *i *as predicted by the prognostic indices MPI and SPI, respectively. It is then clear that the weighted average rank position of sample *i *over the two indices, that is:

riH≡wMriM+wPriP
 MathType@MTEF@5@5@+=feaafiart1ev1aaatCvAUfeBSjuyZL2yd9gzLbvyNv2Caerbhv2BYDwAHbqedmvETj2BSbqee0evGueE0jxyaibaiKI8=vI8tuQ8FMI8Gi=hEeeu0xXdbba9frFj0=OqFfea0dXdd9vqai=hGuQ8kuc9pgc9s8qqaq=dirpe0xb9q8qiLsFr0=vr0=vr0dc8meaabaqaciGacaGaaeqabaqadeqadaaakeaacaWGYbWaa0baaSqaaiaadMgaaeaacaWGibaaaOGaeyyyIORaam4DamaaBaaaleaacaWGnbaabeaakiaadkhadaqhaaWcbaGaamyAaaqaaiaad2eaaaGccqGHRaWkcaWG3bWaaSbaaSqaaiaadcfaaeqaaOGaamOCamaaDaaaleaacaWGPbaabaGaamiuaaaaaaa@42A1@

with *w*_*M *_+ *w*_*P *_= 1 represents on overall relative risk of sample *i *as predicted by both indices. Finally, the D-index of this hybrid prognostic index, HPI, can be evaluated using the same procedure as before.

### The Nottingham Prognostic Index

Because the precise number of positive axillary nodes was not known in two of the external studies (UPP and JRH-2), we used here a slightly different definition for the NPI:

NPI = 0.2 × (*Tumor*_*Size *[cm]) + *Grade *+ 1.5 × (*Node*_*Status*) + 1

where Node Status can be positive (1) or negative (0) and Grade can be 1, 2 or 3. While our modified NPI gave slightly smaller D-index values than the NPI in those cohorts where axillary node information was available, the difference between the two values was minimal, thus validating our definition.

### Software used

All computations were carried out using the *R*-software environment version 2.2.1 [37]. We made use of the *R*-packages survival, mclust and vabayelMix. *R-*scripts that apply the algorithms as implemented here are available on request.

## Additional data files

The following additional data are available with the online version of this paper. Additional data file 1 is a figure showing the weighted average D-index over test sets in the training cohorts. Additional data file 2 is a figure showing the D-index variation as a function of top-ranked genes in the overall molecular classifier. Additional data file 3 lists the 52-gene optimal classifier. Additional data files 4, 5 and 6 are figures showing the D-index of hybrid equal-and-variable weight prognostic models in independent cohorts. Additional data files 7, 8 and 9 contain the GO analysis results for the top 200 prognostic genes, as produced by the GOTM [29]. Additional data file 10 shows the overlap of our 52-gene classifier with the prognostic signatures in [4,10-12]. Additional data file 11 contains the clinical and gene expression data of the NCH cohort.

## Supplementary Material

Additional data file 1 Weighted average D-index over the test sets in the training cohorts as a function of the number of top-ranked genes in the molecular classifier. The corresponding log-rank test p values are shown for the two training cohorts with test sets, NKI2 (blue) and EMC (green), separately. Results are shown for two independent choices of training/test set partitions (realizations) within the training cohorts. Click here for file

Additional data file 2 The weighted average D-index over the three training cohorts NKI2, EMC and NCH is shown as a function of the incremental number of top-ranked genes in the overall molecular classifier. Weights were chosen proportional to the number of samples in each cohort. The ranking of the genes was determined by the global average Cox-score over the ten training-test set partitions and three training cohorts. Click here for file

Additional data file 3 The 52-gene optimal classifier. Click here for file

Additional data file 4 The D-index (and associated 68% CI) of prognostic separation for the hybrid prognostic index (HPI ~ SPI + MPI*; red) in the three external cohorts **(A1) **JRH-1, **(B1) **UPP and **(C1) **JRH-2. In all cases, the risk-ordering of samples by the HPI is determined by the average ranking induced by SPI and MPI*. Also shown is the D-index of the standard prognostic index (BLACK) in each of the three external cohorts. The corresponding log-rank test p values (in log10-space) of the SPI and HPI classifiers are shown for the cohorts **(A2) **JRH-1, **(B2) **UPP and **(C2) **JRH-2. The 0.05 confidence threshold line is indicated (green). Click here for file

Additional data file 5 The mean D-index and associated standard errors of prognostic separation for the 10 hybrid prognostic (HPI_*p*_; red) indices in the three external cohorts **(A1) **JRH-1, **(B1) **UPP and **(C1) **JRH-2. In all cases, the risk-ordering of samples by the HPI_*p *_is determined by the average ranking induced by SPI and MPI_*p*_. Also shown is the D-index of the pathological/classical prognostic index (black) in each of the three external cohorts. The corresponding log-rank test p values (in log10-space) of the SPI and HPI_*p *_classifiers are shown for the cohorts **(A2) **JRH-1, **(B2) **UPP and **(C2) **JRH-2. Click here for file

Additional data file 6 The mean D-index and associated standard errors over the ten hybrid prognostic classifiers (HPI_*p*_) in the three external independent cohorts. The D-index of the HPI_*p *_is parameterized by the weight *w*_*M *_given to MPI_*p*_. Thus, for *w*_*M *_= 0 we have a pure histopathological classifier, while for *w*_*M *_= 1 the prognostic index HPI_*p *_= MPI_*p*_. It turns out that, because of the relatively small number of samples (36) with available node status and NPI information in JRH-2, there were weight values for which the D-index became too large for graphical representation. Click here for file

Additional data file 7 Gene ontology analysis results for the top 200 prognostic genes. Click here for file

Additional data file 8 Gene ontology analysis results for the top 200 prognostic genes. Click here for file

Additional data file 9 Gene ontology analysis results for the top 200 prognostic genes. Click here for file

Additional data file 10 Overlap of our 52-gene classifier with the prognostic signatures in [4,10-12]. Click here for file

Additional data file 11 Clinical and gene expression data of the NCH cohort. Click here for file

## Figures and Tables

**Table 1:** Breast cancer data sets used

Study	Cohort name	Platform	ER+ samples	Events (RIP/DM)
van de Vijver [18]	NKI2	oligos Agilent	226	45
Wang [11]	EMC	oligos Affymetrix	208	80
Naderi [12]	NCH	oligos Agilent	93	21
Sotiriou [25]	JRH-1	spotted cDNA	65	20
Miller [26]	UPP	oligos Affymetrix	213	49
Sotiriou [4]	JRH-2	oligos Affymetrix	72	17

Study, cohort name, microarray platform, number of ER+ patients and death (or surrogate distant metastasis) events among ER+ cases. The cohorts are described in [4,11,12,18,25,26].

**Table 2:** The D-index of prognostic factors across cohorts

	Training			Test		
Factor	NKI2	ECM	NCH	JRH-1	UPP	JRH-2*

Grade	3.80 (<10^-5^)	NA	3.57 (0.001)	3.84 (0.003)	2.55 (0.0003)	2.15 (0.17)
Node status	1.01 (0.97)	all LN-	2.23 (0.05)	2.64 (0.04)	4.03 (<10^-6^)	2.36 (0.25)
Size	1.59 (0.06)	NA	3.36 (0.003)	4.16 (<10^-3^)	3.18 (<10^-5^)	3.04 (0.008)
NPI	2.27 (<10^-3^)	NA	4.07 (<10^-3^)	5.16 (<10^-4^)	3.82 (<10^-7^)	3.78 (0.03)
MPI^†^	3.32 (<10^-3^)	2.29 (0.002)	NA	3.20 (0.002)	2.71 (<10^-4^)	7.96 (<10^-4^)
MPI^‡^	3.64 (<10^-7^)	2.56 (<10^-6^)	6.45 (<10^-5^)	3.44 (<10^-3^)	2.80 (<10^-4^)	11.26 (<10^-5^)
MPI^§^	3.64 (<10^-6^)	2.51 (<10^-5^)	6.51 (<10^-5^)	3.10 (<10^-5^)	2.84 (0.001)	10.10 (<10^-4^)

For the classical prognostic factors we give, where available, the D-index and log-rank test p values in the training cohorts NKI2, ECM and NCH, and test cohorts JRH-1, UPP and JRH-2. *For JRH-2 the number of samples with available grade and node status information were only 57 and 38, respectively. ^†^For the MPI we give the median D-index and log-rank test p value over the ten molecular classifiers. The range for the D-index and p values over the 10 classifiers were: 2.27 to 4.35 (0.009 to 1.1 × 10^-5^) in NKI2; 1.78 to 2.75 (0.024 to 2 × 10^-4^) in ECM; 2.04 to 3.96 (0.039 to 0.0003) in JRH-1; 2.39 to 3.04 (1.7 × 10^-4 ^to 6.7 × 10^-6^) in UPP; and 5.08 to 12.61 (8 × 10^-4 ^to 8.4 × 10^-6^) in JRH-2. ^‡^The MPI based on the optimal 52-gene classifier. ^§^The MPI based on the 17-gene classifier. NA, not available.

**Table 3:** Top prognostic genes in ER+ breast cancer

UniGene symbol	Coefficient sign	Cytoband	GO
RACGAP1	+	12q13.12	GTPase activator activity, electron transporter activity
STK6	+	20q13.2-q13.3	ATP binding, mitosis, phosphorylation, kinase activity
HUMMLC2B	-	16p11.2	calcium ion binding, muscle myosin
MELK	+	9p13.2	ATP binding, phosphorylation, tyrosine kinase activity
PPARA	-	22q12-q13.1	Transcription factor, steroid hormone activity/lipid metabolism
DHCR7	+	11q13.2-q13.5	cholesterol binding and biosynthesis, electron transporter activity
MAD2L1	+	4q27	Cell-cycle, mitotic checkpoint, spindle
ZWINT	+	10q21-q22	Nucleus
KIF20A	+	5q31	ATP binding, microtubule associated complex
CDCA8	+	1p34.3	Cytokinesis
KIAA0101	+	15q22.31	*PCNA *associated factor
TIMELESS	+	12q12-q13	Development, negative regulation of transcription
PTTG1	+	5q35.1	DNA metabolism, repair, replication and chromosome cycle
WSB2	+	12q24.23	Intracellular signaling cascade
ABCC5	+	3q27	ATP binding, ATPase activity, transmembrane movement
KIF23	+	15q23	ATP binding, microtubule complex/motor activity, mitosis
H2AFY	+	5q31.3-q32	DNA binding, chromosome organization, nucleosome assembly

Top ranked 17 prognostic genes in ER+ breast cancer as determined by a meta-analysis of three major breast cancer data sets. We give the sign of their global average Cox-regression coefficient ('+' means upregulated in poor outcome tumors; '-' means downregulated in poor outcome tumors), cytoband position and selected abbreviated Gene Ontology.

**Table 4:** Multivariate D-index analysis

	1	2	3	4	5	6	7	8	9	10	Opt.
JRH-1											
MPI (A)	0.21	0.19	0.03	0.03	0.05	0.06	0.43	0.12	0.04	0.16	0.15
Grade	0.06	0.07	0.11	0.05	0.10	0.11	0.05	0.08	0.10	0.10	0.05
Node status	0.79	0.73	0.96	0.86	0.93	0.89	0.65	0.87	0.83	0.91	0.91
Size	0.07	0.01	0.02	0.05	0.03	0.05	0.04	0.02	0.02	0.11	0.11
MPI (B)	0.22	0.31	0.08	0.06	0.13	0.14	0.42	0.18	0.11	0.16	0.16
NPI	<0.005	<0.005	<0.005	<0.005	<0.005	0.01	<0.005	<0.005	<0.005	0.01	0.01
											
UPP											
MPI (A)	0.01	0.01	0.06	0.01	0.02	0.01	<0.005	0.01	0.01	0.01	0.01
Grade	0.72	0.84	0.85	0.90	0.98	0.99	0.73	0.83	0.85	0.88	0.83
Node status	<0.005	<0.005	<0.005	<0.005	<0.005	<0.005	<0.005	<0.005	<0.005	<0.005	<0.005
Size	0.02	0.02	0.02	0.02	0.02	0.02	0.02	0.03	0.02	0.02	0.02
MPI (B)	0.02	0.03	0.12	0.02	0.04	0.03	0.01	0.02	0.02	0.02	0.02
NPI	<0.005	<0.005	<0.005	<0.005	<0.005	0.01	<0.005	<0.005	<0.005	<0.005	<0.005
											
JRH-2											
MPI (A)	0.01	0.01	0.01	0.01	0.02	0.01	0.01	0.04	0.01	<0.005	0.01
Grade	0.19	0.10	0.24	0.24	0.07	0.15	0.23	0.13	0.21	0.43	0.10
Node status	0.44	0.16	0.76	0.45	0.24	0.35	0.67	0.50	0.25	0.37	0.24
Size	0.12	0.73	0.28	0.28	0.93	0.82	0.27	0.47	0.35	0.36	0.97
MPI (B)	<0.005	<0.005	0.01	0.01	<0.005	0.01	<0.005	0.01	<0.005	<0.005	<0.005
NPI*	0.51	0.53	0.75	0.58	0.97	0.78	0.99	0.63	0.50	0.47	0.96

Given are the rounded p values (to two significant digits) of the D-indices for two multivariate models, model A is *log*(*h*(*t*)) ~ (*Grade*) + (*NodeStatus*) + (*TumorSize*) + *MPI*_*p *_and model-B is *log*(*h*(*t*)) ~ *NPI *+ *MPI*_*p*_, in the three external cohorts JRH-1, UPP and JRH-2. Columns label the 10 different derived molecular classifiers, depending on the training-test set partition *p *used, and the optimal 52-gene classifier. *For JRH-2 only 36 samples with *NPI *information were available. Opt., optimal.

**Table 5:** The prognostic added value of the MPI

Model	JRH-1	UPP	JRH-2^†^
Grade	3.85	2.55	2.15
Grade + MPI*	5.85 (2.49-13.72)	2.85 (1.79-4.51)	10.60 (2.79-40.20)
Grade + MPI**	4.62 (2.15-9.91)	2.90 (1.83-4.58)	8.14 (2.11-31.31)
Node Status	2.64	4.03	2.36
Node Status + MPI*	2.98 (1.48-6.01)	4.71 (2.83-7.86)	14.07 (2.08-94.84)
Node Status + MPI**	3.09 (1.53-6.23)	4.40 (2.74-7.06)	11.79 (1.86-74.44)
Size	4.16	3.18	3.04
Size + MPI*	5.40 (2.51-11.62)	3.41 (2.16-5.38)	5.21 (2.29-11.84)
Size + MPI**	4.88 (2.35-10.13)	3.65 (2.29-5.81)	4.51 (2.08-9.77)
NPI	5.16	3.82	3.78
NPI + MPI*	5.85 (2.54-13.47)	4.02 (2.52-6.41)	19.11 (2.62-139.3)
NPI + MPI**	4.93 (2.30-10.56)	4.04 (2.53-6.44)	25.23 (2.53-251.6)

For each standard prognostic index *SPI *(grade, node status, size and *NPI*) we compare their D-index with the D-index of the corresponding equal-weight hybrid prognostic model, defined by a hybrid prognostic index *HPI*, where *HPI *~ *SPI *+ MPI* or *HPI *~ *SPI *+ MPI** (see Materials and methods). 95% CI for the hybrid prognostic model D-index values are shown in brackets. MPI* denotes the index of the optimal 52-gene classifier. MPI** denotes the index of the 17-gene classifier. ^†^For JRH-2 only 36 samples with *NPI *information were available.
