# Supplementary figures and images for: A consensus prognostic gene expression classifier for ER positive breast cancer
Source: Genome Biol. 2006 Oct 31;7(10):R101. doi: 10.1186/gb-2006-7-10-r101 (PMC1794561; doi:10.1186/gb-2006-7-10-r101)

A1)

Realisation-1

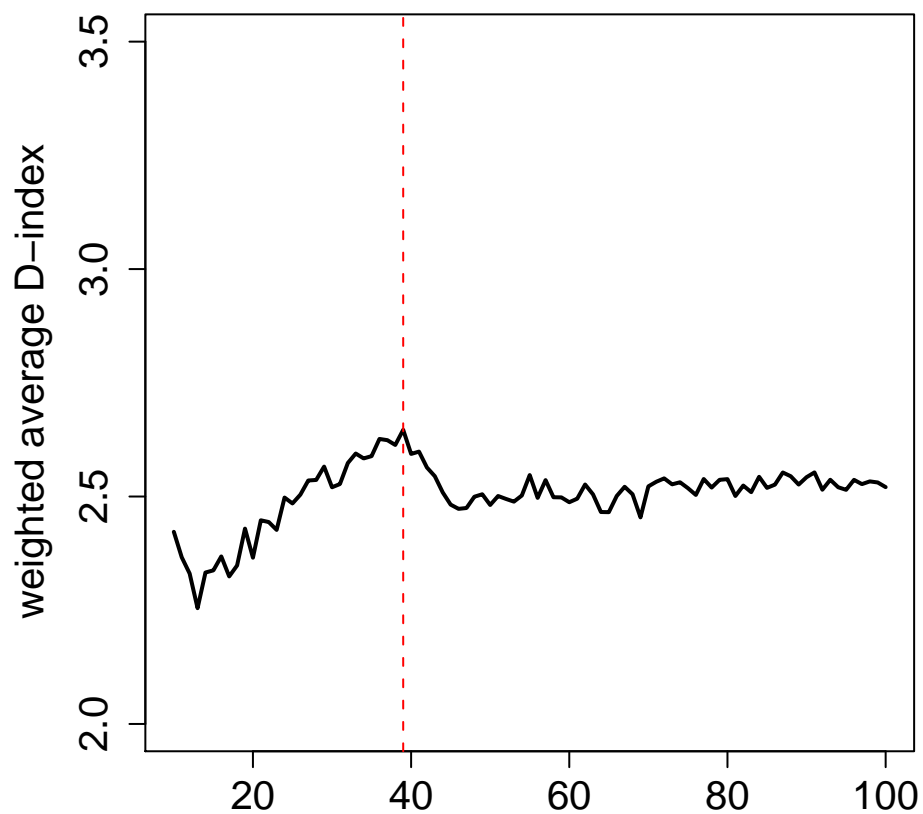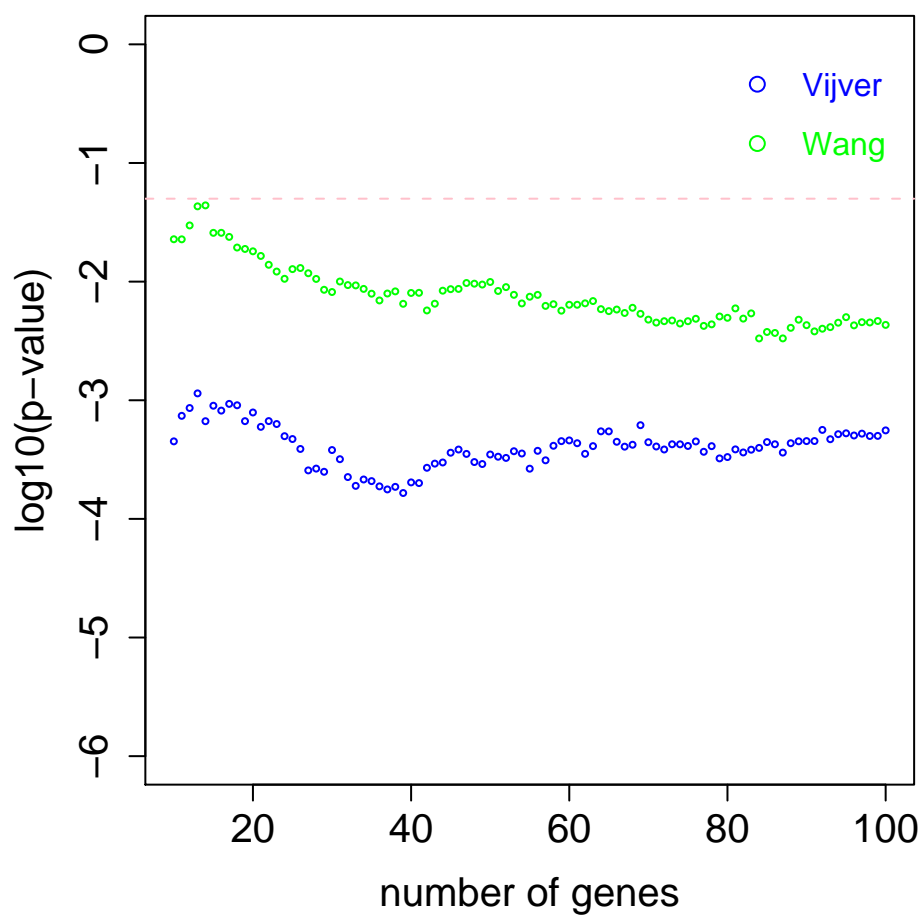

A2)

Realisation-8

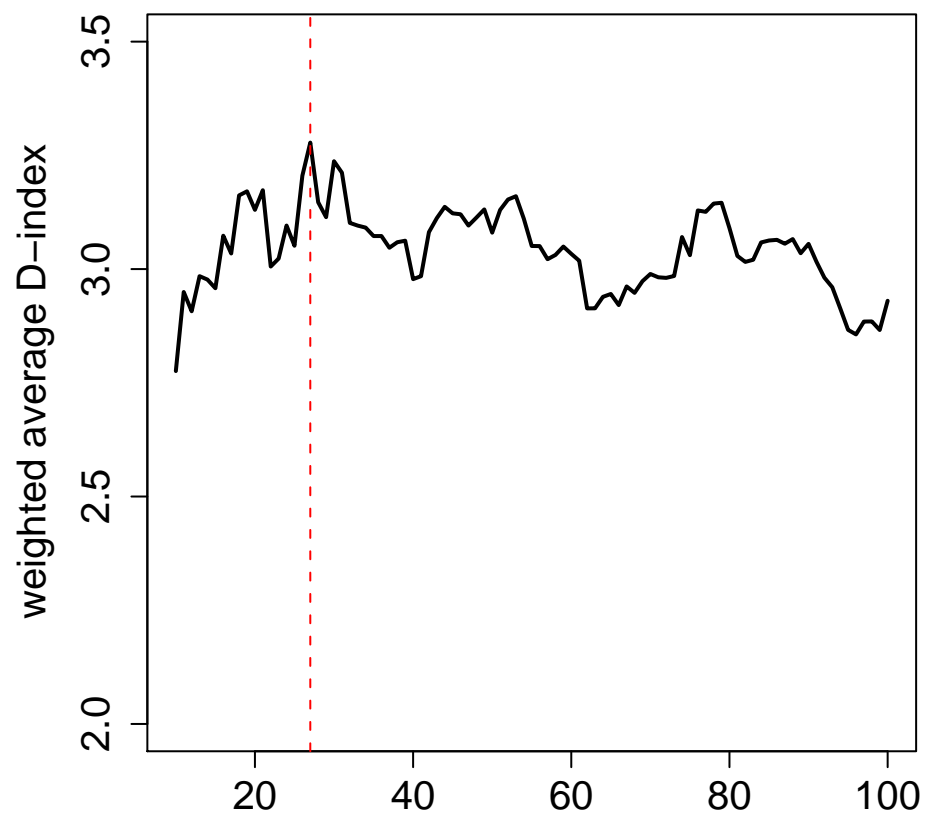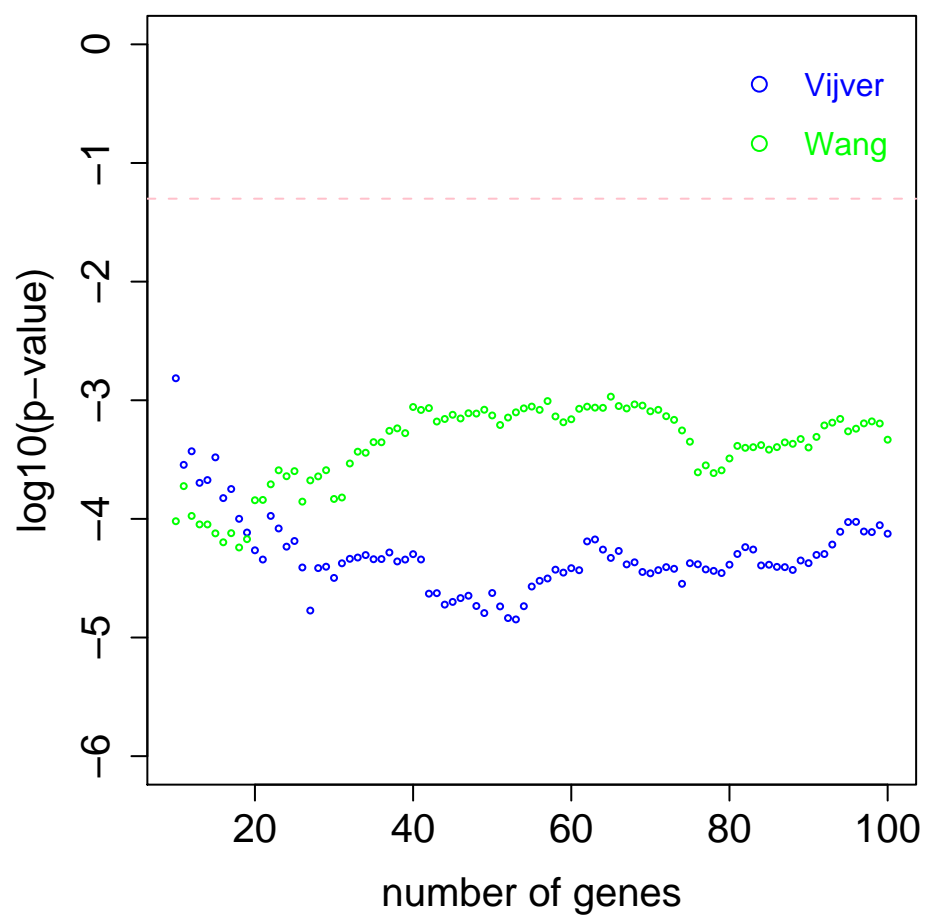

Supplement: Additional data file 1 — Weighted average D-index over the test sets in the training cohorts as a function of the number of top-ranked genes in the molecular classifier. The corresponding log-rank test p values are shown for the two training cohorts with test sets, NKI2 (blue) and EMC (green), separately. Results are shown for two independent choices of training/test set partitions (realizations) within the training cohorts. [file gb-2006-7-10-r101-S1.pdf]

Weighted average D-index over training cohorts

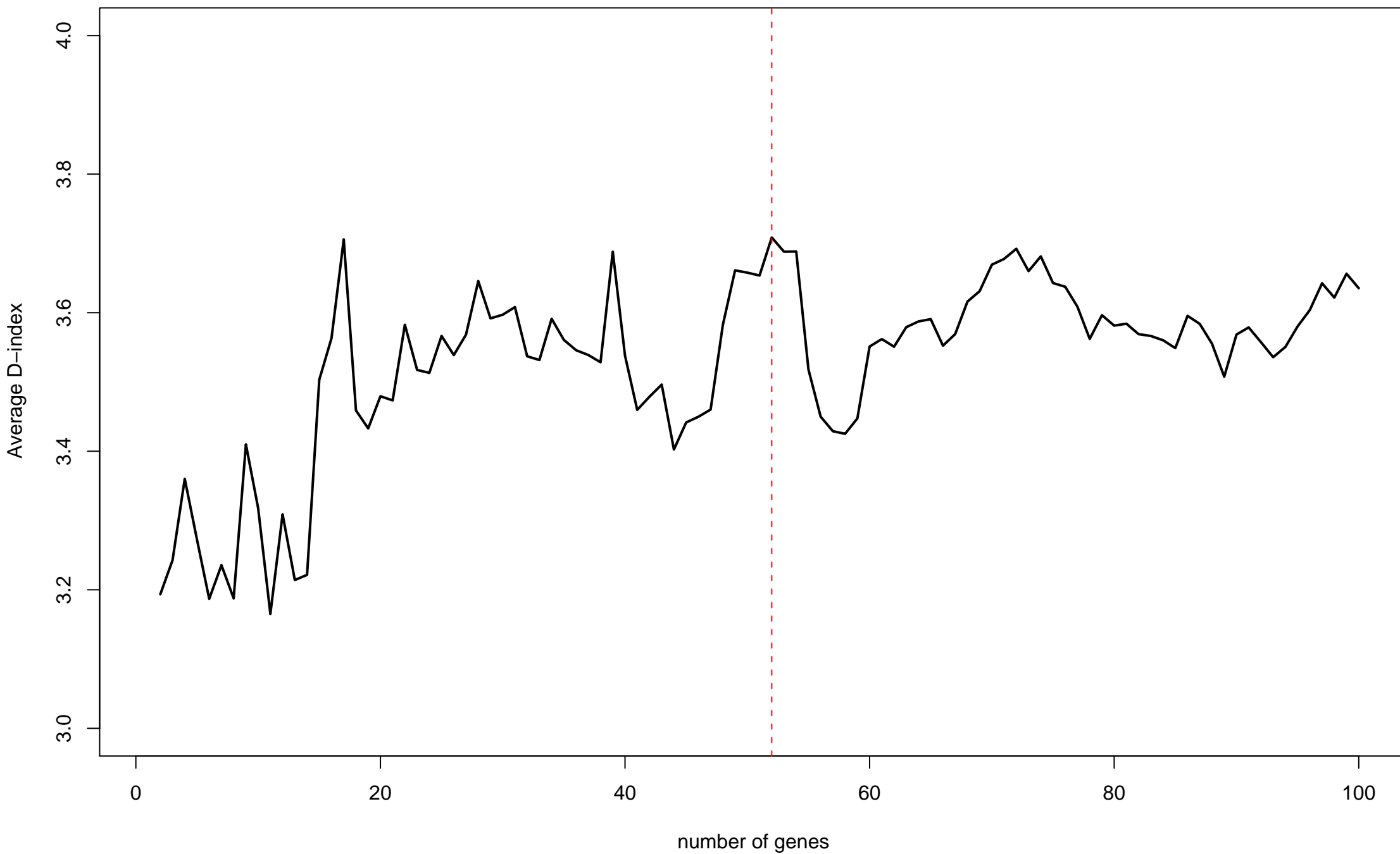

Supplement: Additional data file 2 — The weighted average D-index over the three training cohorts NKI2, EMC and NCH is shown as a function of the incremental number of top-ranked genes in the overall molecular classifier. Weights were chosen proportional to the number of samples in each cohort. The ranking of the genes was determined by the global average Cox-score over the ten training-test set partitions and three training cohorts. [file gb-2006-7-10-r101-S2.pdf]

A1)

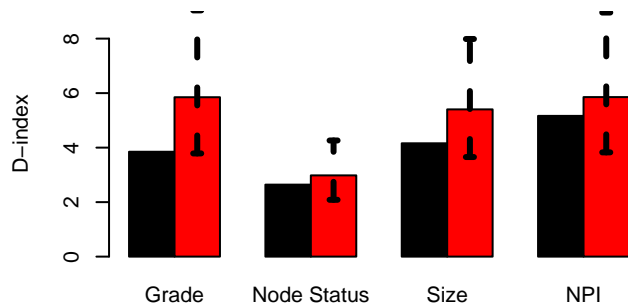

A2)

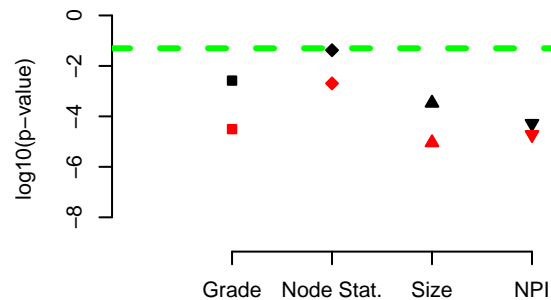

B1)

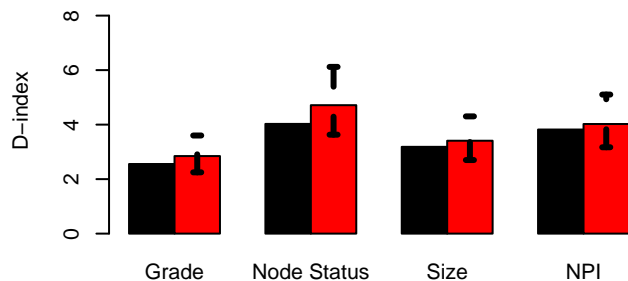

B2)

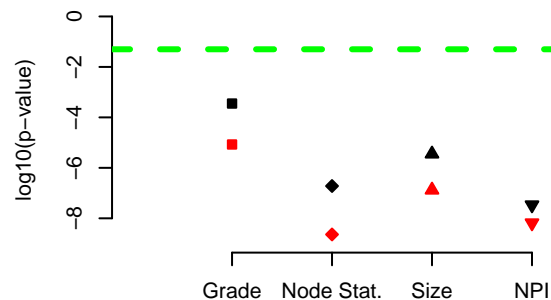

C1)

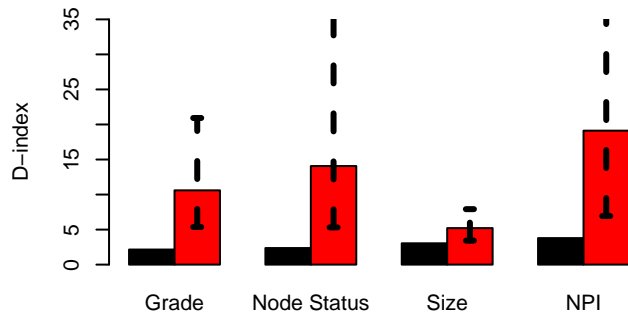

C2)

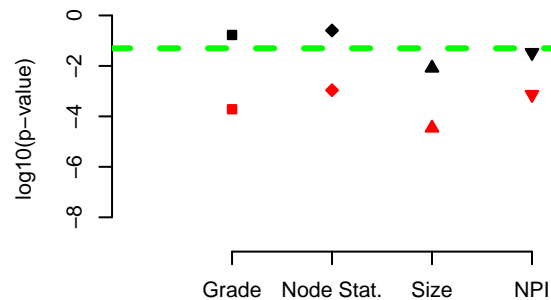

Supplement: Additional data file 4 — The D-index (and associated 68% CI) of prognostic separation for the hybrid prognostic index (HPI ~ SPI + MPI*; red) in the three external cohorts (A1) JRH-1, (B1) UPP and (C1) JRH-2. In all cases, the risk-ordering of samples by the HPI is determined by the average ranking induced by SPI and MPI*. Also shown is the D-index of the standard prognostic index (BLACK) in each of the three external cohorts. The corresponding log-rank test p values (in log10-space) of the SPI and HPI classifiers are shown for the cohorts (A2) JRH-1, (B2) UPP and (C2) JRH-2. The 0.05 confidence threshold line is indicated (green). [file gb-2006-7-10-r101-S4.pdf]

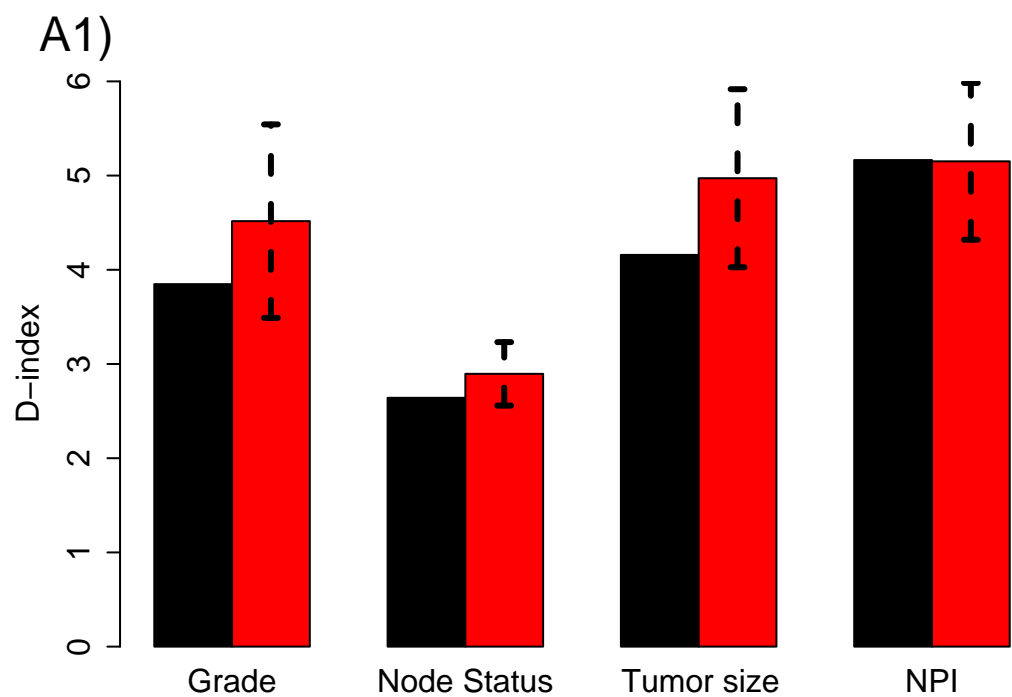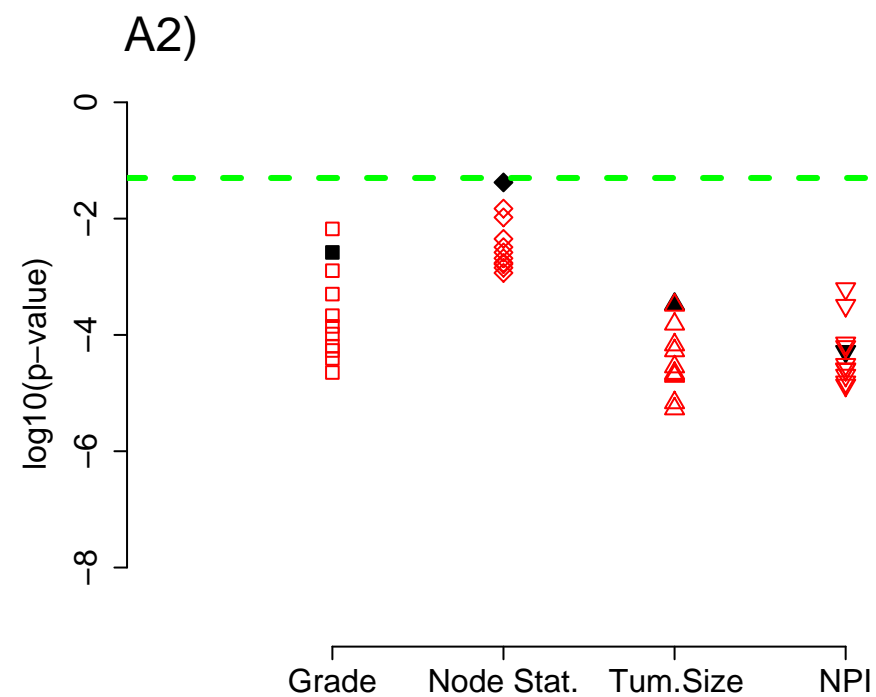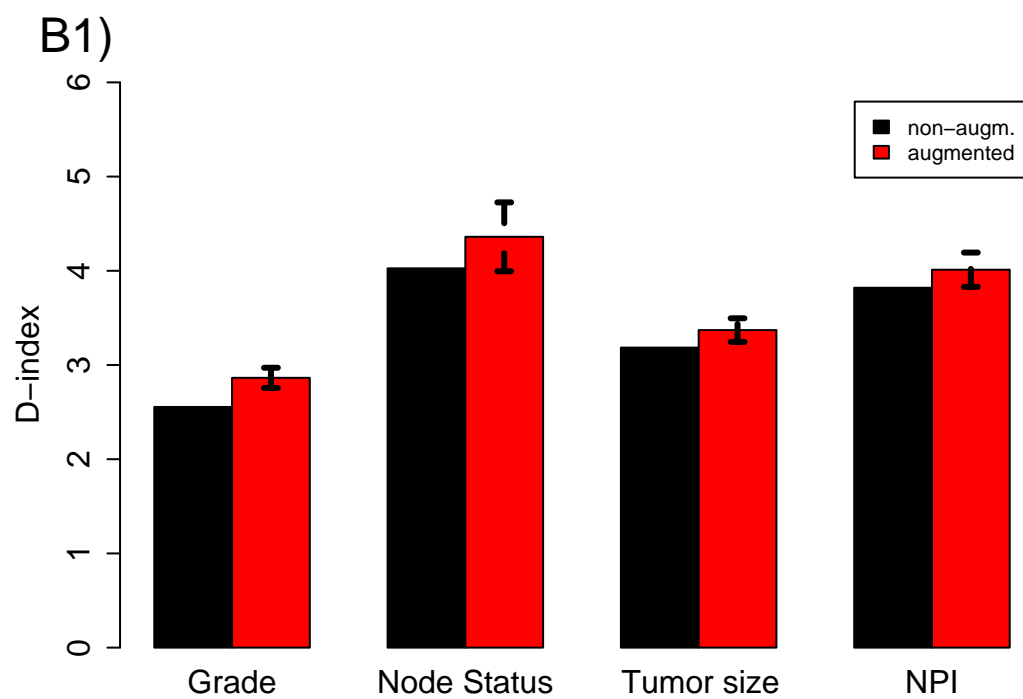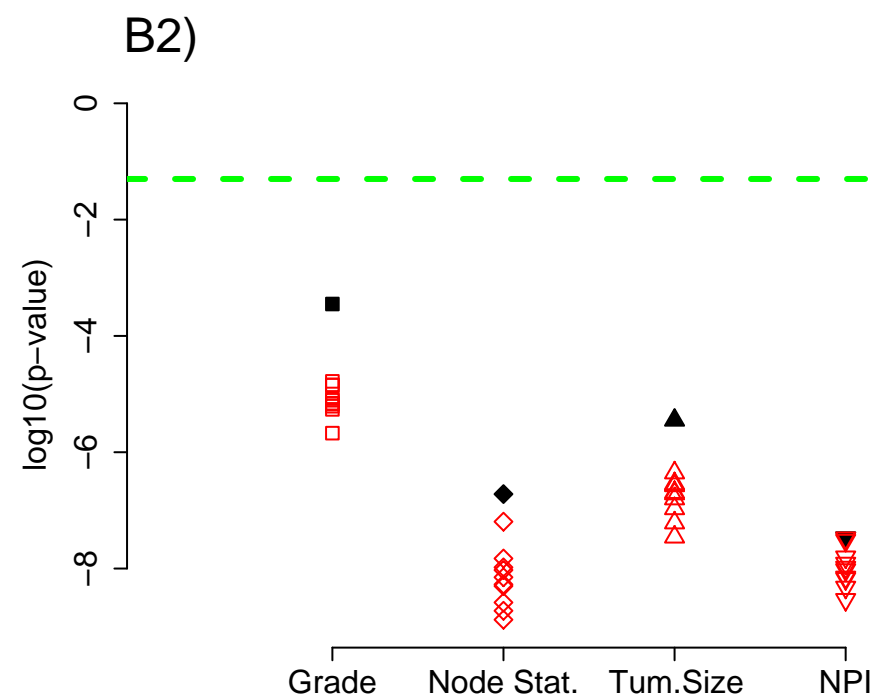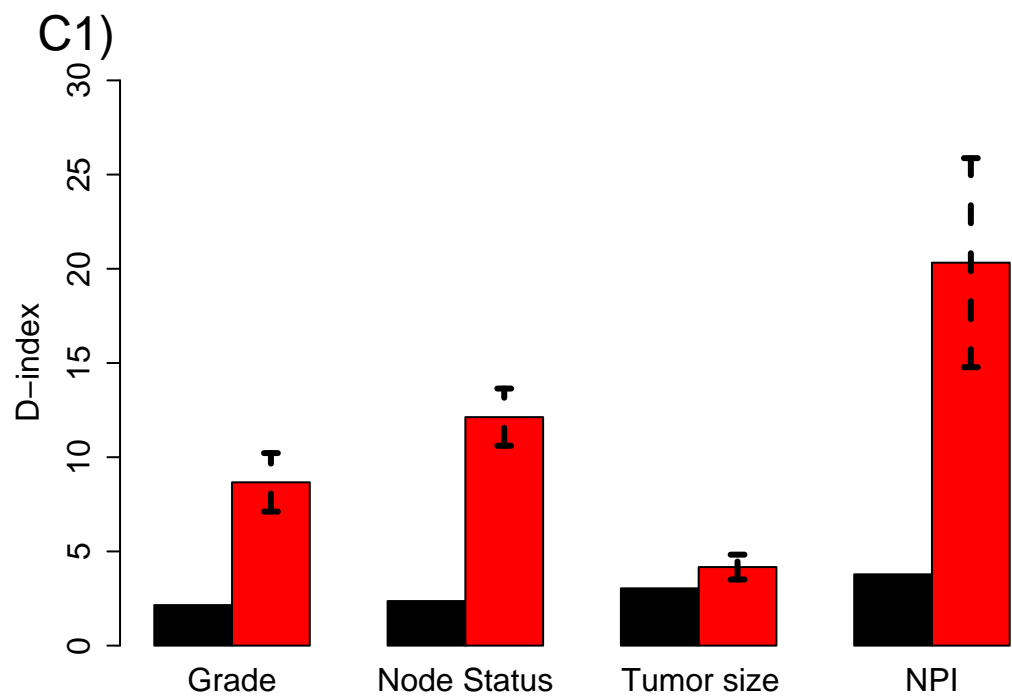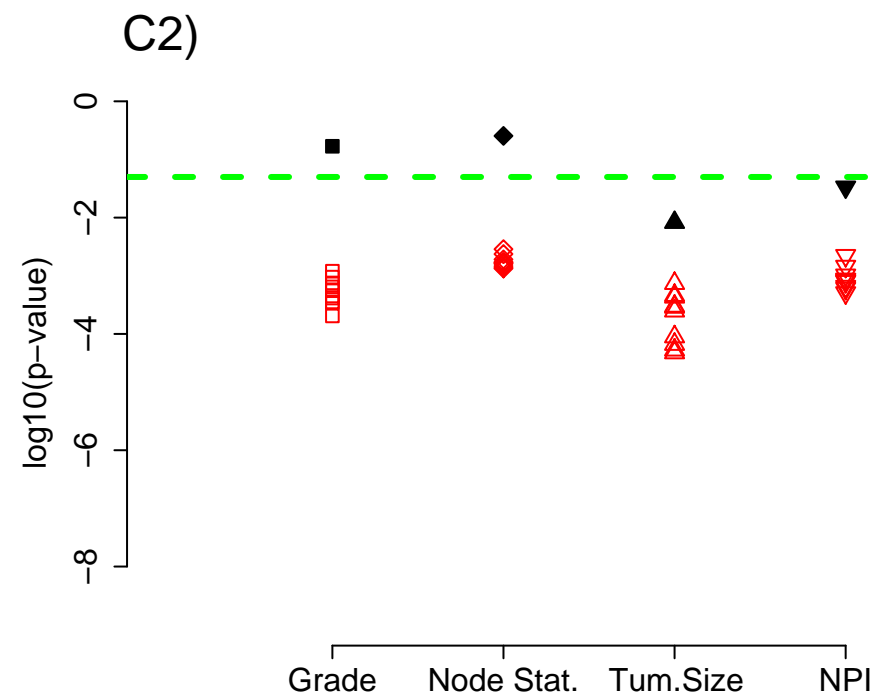

Supplement: Additional data file 5 — The mean D-index and associated standard errors of prognostic separation for the 10 hybrid prognostic (HPIp; red) indices in the three external cohorts (A1) JRH-1, (B1) UPP and (C1) JRH-2. In all cases, the risk-ordering of samples by the HPIp is determined by the average ranking induced by SPI and MPIp. Also shown is the D-index of the pathological/classical prognostic index (black) in each of the three external cohorts. The corresponding log-rank test p values (in log10-space) of the SPI and HPIp classifiers are shown for the cohorts (A2) JRH-1, (B2) UPP and (C2) JRH-2. [file gb-2006-7-10-r101-S5.pdf]

**JRH-1: Grade**

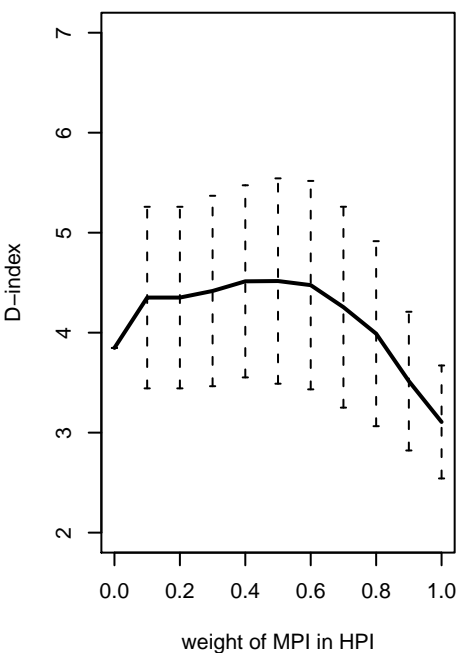

**JRH-1: Size**

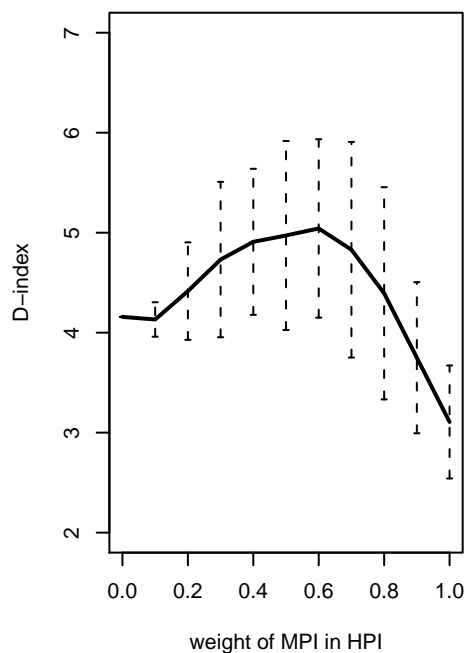

**JRH-1: Stage**

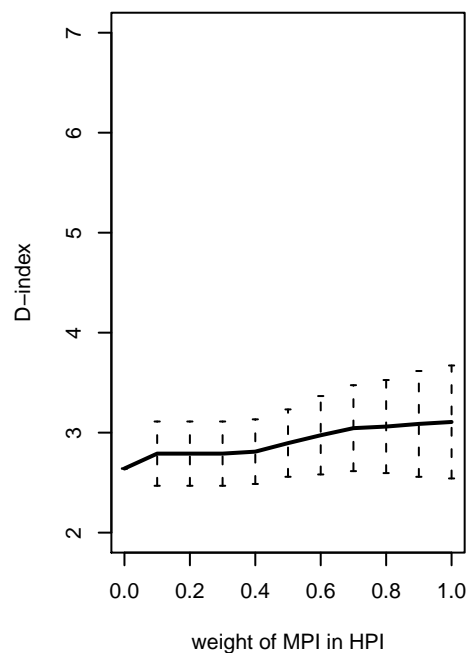

**JRH-1: NPI**

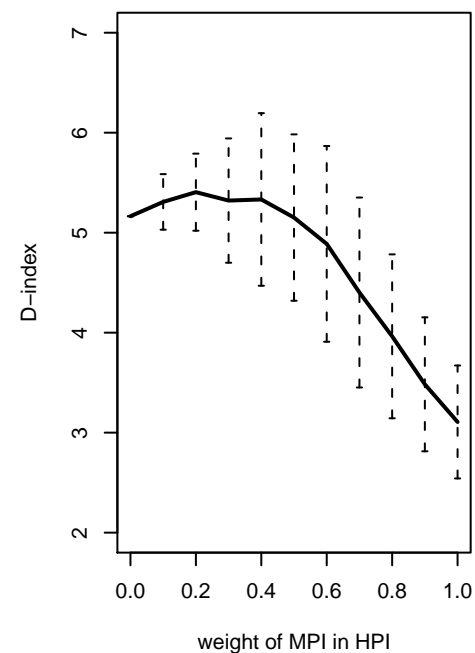

**UPP.: Grade**

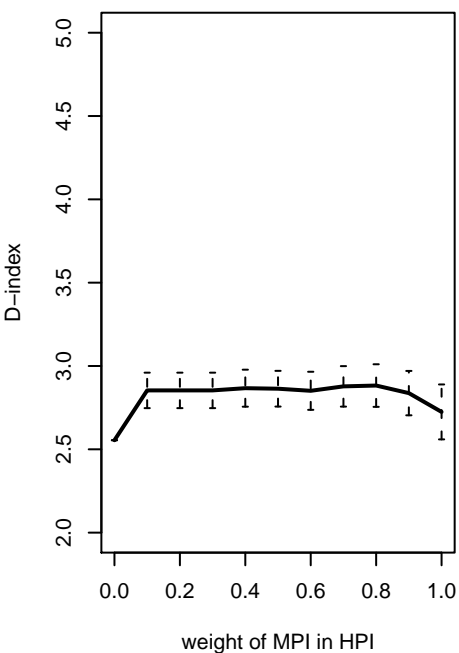

**UPP.: Size**

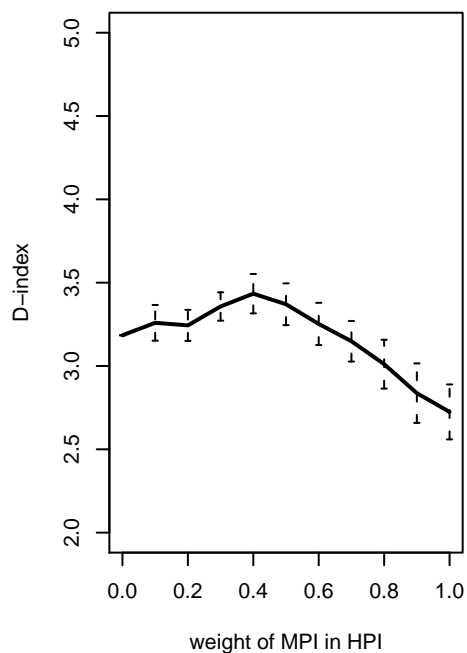

**UPP.: Stage**

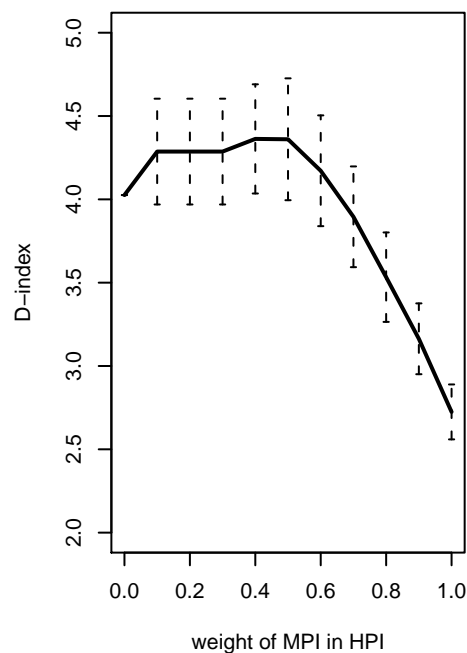

**UPP.: NPI**

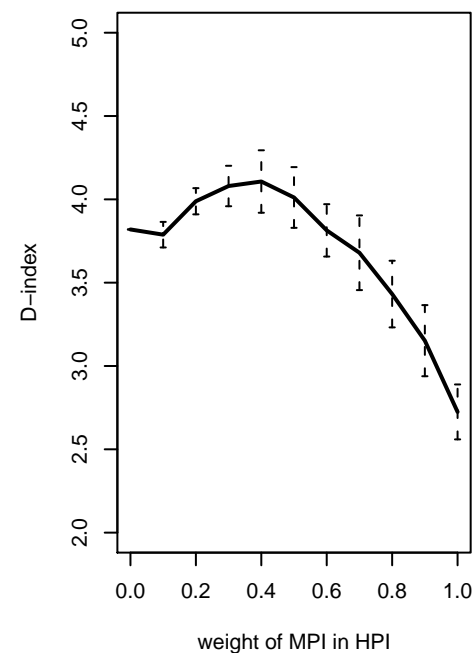

**JRH-2: Grade**

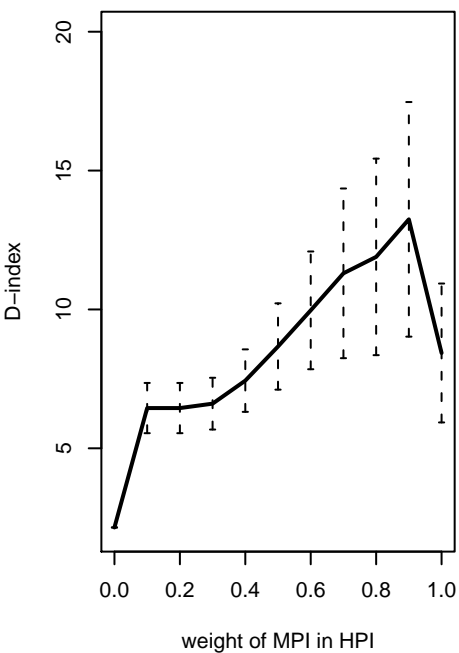

**JRH-2: Size**

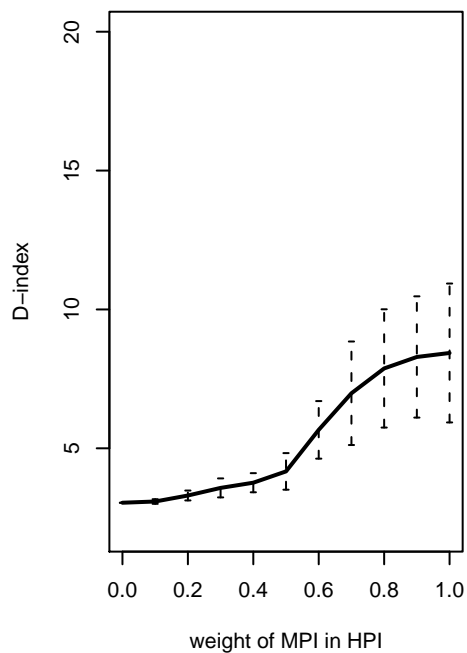

Supplement: Additional data file 6 — The mean D-index and associated standard errors over the ten hybrid prognostic classifiers (HPIp) in the three external independent cohorts. The D-index of the HPIp is parameterized by the weight wM given to MPIp. Thus, for wM = 0 we have a pure histopathological classifier, while for wM = 1 the prognostic index HPIp = MPIp. It turns out that, because of the relatively small number of samples (36) with available node status and NPI information in JRH-2, there were weight values for which the D-index became too large for graphical representation. [file gb-2006-7-10-r101-S6.pdf]

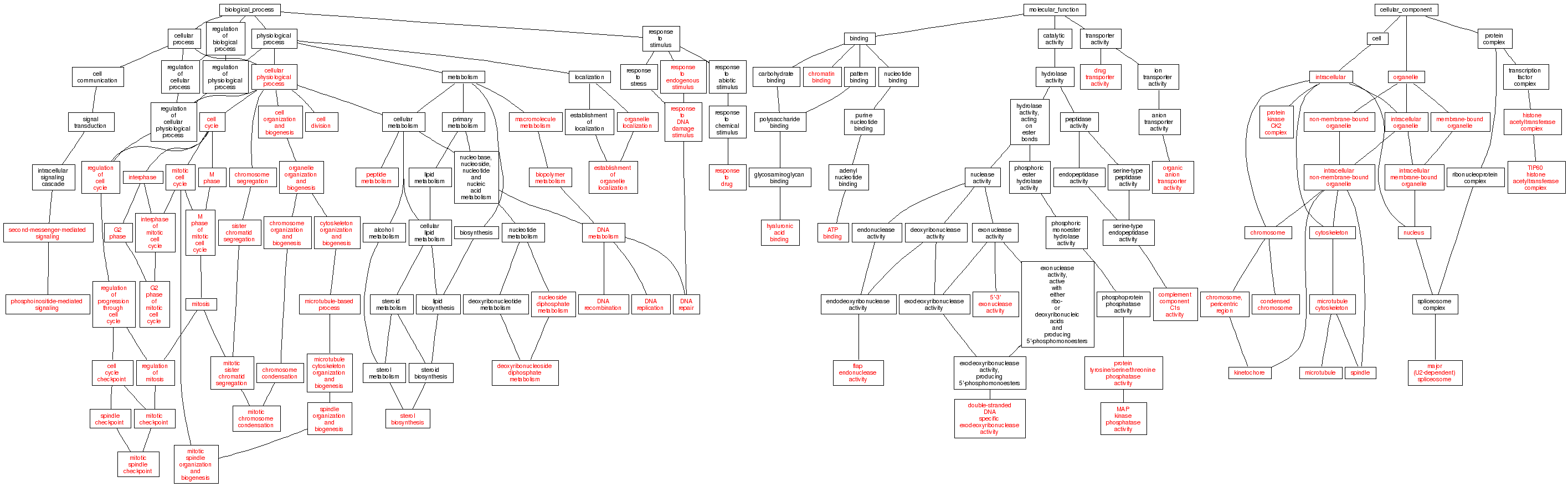

Supplement: Additional data file 7 — Gene ontology analysis results for the top 200 prognostic genes. [file gb-2006-7-10-r101-S7.gif]
