# Supplementary material for: A consensus prognostic gene expression classifier for ER positive breast cancer
Source: Genome Biol. 2006 Oct 31;7(10):R101. doi: 10.1186/gb-2006-7-10-r101 (PMC1794561; doi:10.1186/gb-2006-7-10-r101)
Supplement: Additional data file 3 — The 52-gene optimal classifier. [file gb-2006-7-10-r101-S3.pdf]

Additional Data File-3 - The optimal 52-gene molecular classifier in ER+ breast cancer

Top ranked 52 prognostic genes in ER+ breast cancer as determined by a meta-analysis of three major breast cancer data sets. We give the sign of their global average Cox-regression coefficient ("+" means upregulated in poor outcome tumors, "-" means downregulated in poor outcome tumors), cytoband position and selected abbreviated Gene Ontology.

UniGene Symbol	Coeff.Sign	Cytoband	GOabr
RACGAP1	+	12q13.12	GTPase activator activity—electron transporter activity
STK6	+	20q13.2−q13.3	ATP binding—mitosis—phosphorylation—kinase activity
HUMMLC2B	−	16p11.2	calcium ion binding—muscle myosin
MELK	+	9p13.2	ATP binding—phosphorylation—tyrosine kinase activity
PPARA	−	22q12-q13.1	Transcription factor—Steroid hormone activity/lipid metabolism
DHCR7	+	11q13.2−q13.5	cholesterol binding & biosynthesis—electron transporter activity
MAD2L1	+	4q27	Cell-cycle—mitotic checkpoint—spindle
ZWINT	+	10q21−q22	nucleus
KIF20A	+	5q31	ATP binding—microtubule associated complex
CDCA8	+	1p34.3	cytokinesis
KIAA0101	+	15q22.31	*PCNA* associated factor
TIMELESS	+	12q12−q13	development—negative regulation of transcription
PTTG1	+	5q35.1	DNA metabolism,repair,replication and chromosome cycle
WSB2	+	12q24.23	intracellular signaling cascade
ABCC5	+	3q27	ATP binding—ATPase activity—transmembrane movement
KIF23	+	15q23	ATP binding—microtubule complex/motor activity—mitosis
H2AFY	+	5q31.3−q32	DNA binding—chromosome organization—nucleosome assembly
BIRC5	+	17q25	G2/M transition—anti−apoptosis—microtubule binding
ESPL1	+	12q	apoptosis—chromosome segregation—regulation of cell cycle
ZMYND11	−	10p14	DNA binding—cell cycle—cell proliferation
SPAG5	+	17q11.2	cell cycle—cytokinesis—mitosis
DDX39	+	19p13.12	ATP binding—helicase/hydrolase activity
ATAD2	+	8q24.13	ATP binding
CFDP1	+	16q22.2−q22.3	regulation of transcription-chromating remodelling.
TGFBR3	−	1p33−p32	development—transf. growth factor beta receptor signaling pathway
LMNB1	+	5q23.3−q31.1	lamin filament—nucleus—structural molecule activity
CCNE2	+	8q22.1	cell cycle checkpoint—cytokinesis—regulation of cell cycle
SNFT	−	1q32.3	regulation of transcription
ZNF198	+	13q11-q12	regulation of transcription
RAD54L	+	1p32	ATP binding—DNA binding——DNA repair—meiosis
SIN3B	+	19p13.11	nucleus—regulation of transcription
FLJ20641	+	12q23.3	UvrD helicase
PIP5K3	+	2q34	ATP binding—intracellular signaling cascade—kinase activity
PSMD7	+	16q23−q24	proteasome regulatory particle (sensu Eukaryota)
BUB1B	+	15q15	cell proliferation—mitotic checkpoint—phosphorylation
EZH2	+	7q35−q36	chromatin architecture—regulation of transcription
SLCO1B1	+	12p	ion transport—transporter activity
FLJ10292	+	12p13.2	nucleus—sex determination, microtubule cytoskeleton
TCEB1	+	8q21.11	protein binding—Ubiqutin cycle—transcription
BM039	+	16q23.2	uncharacterised bone marrow protein
CDKN3	+	14q22	cell cycle arrest—negative regulation of cell proliferation
E2F1	+	20q11.2	G1 phase of mitotic cell cycle—apoptosis—cell proliferation
UBE2C	+	20q13.12	positive regulation of cell proliferation—ubiquitin cycle
CDC2	+	10q21.1	cyclin−dependent kinase activity—mitosis—phosphorylation
XPOT	+	12q14.2	binding—nucleus—tRNA binding—transport
NOC4	+	16q24	mitochondrion—molecular function unknown—nucleus
SQLE	+	8q24.1	electron transport—metabolism—oxidoreductase activity
PRAME	+	22q11.22	pref.expressed antigen melanoma
TFAP2B	−	6p21−p12	neurogenesis—transcription factor activity
MYBL2	+	20q13.1	anti−apoptosis—chromatin—development—cell cycle
SFRS15	+	21q22.1	RNA binding—nucleus
FUT9	+	6q16	transferase activity—carbohydrate metabolism
