# Supplementary material for: A consensus prognostic gene expression classifier for ER positive breast cancer
Source: Genome Biol. 2006 Oct 31;7(10):R101. doi: 10.1186/gb-2006-7-10-r101 (PMC1794561; doi:10.1186/gb-2006-7-10-r101)
Supplement: Additional data file 8 — Gene ontology analysis results for the top 200 prognostic genes. [file gb-2006-7-10-r101-S8.html]

Tree view of Enriched GO Categories

**Gene numbers in 62 GO categories were relatively enriched (p<0.01)**  
**O:**Observed gene number in the GO category; **E:**Expected gene number in the GO category;  
**R:**Ratio of enrichment for the GO category; **P:**Significance of enrichment for the GO category  
  

01---biological\_process

02------cellular process

03---------cell communication

04------------signal transduction

05---------------intracellular signaling cascade

06------------------second-messenger-mediated signaling(O=8;E=1.34;R=5.97;P=4.39013433635E-05)

07---------------------phosphoinositide-mediated signaling(O=7;E=0.63;R=11.11;P=1.94749445693E-06)

03---------cellular physiological process(O=67;E=58.7;R=1.14;P=0.00459957343727)

04------------cell cycle(O=23;E=5.4;R=4.26;P=7.3632078069E-10)

05---------------interphase(O=5;E=0.51;R=9.8;P=0.000122920200122)

06------------------interphase of mitotic cell cycle(O=5;E=0.51;R=9.8;P=0.000122920200122)

05---------------M phase(O=14;E=1.52;R=9.21;P=1.14354158563E-10)

06------------------M phase of mitotic cell cycle(O=11;E=1.3;R=8.46;P=3.60193701196E-08)

07---------------------mitosis(O=11;E=1.26;R=8.73;P=2.57254603932E-08)

08------------------------regulation of mitosis(O=5;E=0.37;R=13.51;P=2.41940019371E-05)

09---------------------------mitotic checkpoint(O=3;E=0.16;R=18.75;P=0.000382494856421)

05---------------mitotic cell cycle(O=15;E=1.73;R=8.67;P=5.99335349036E-11)

06------------------interphase of mitotic cell cycle(O=5;E=0.51;R=9.8;P=0.000122920200122)

06------------------M phase of mitotic cell cycle(O=11;E=1.3;R=8.46;P=3.60193701196E-08)

07---------------------mitosis(O=11;E=1.26;R=8.73;P=2.57254603932E-08)

08------------------------regulation of mitosis(O=5;E=0.37;R=13.51;P=2.41940019371E-05)

09---------------------------mitotic checkpoint(O=3;E=0.16;R=18.75;P=0.000382494856421)

05---------------regulation of progression through cell cycle(O=13;E=3.82;R=3.4;P=7.86562941475E-05)

06------------------cell cycle checkpoint(O=4;E=0.41;R=9.76;P=0.000643149899572)

07---------------------mitotic checkpoint(O=3;E=0.16;R=18.75;P=0.000382494856421)

06------------------regulation of cyclin dependent protein kinase activity(O=3;E=0.45;R=6.67;P=0.00978469831304)

06------------------regulation of mitosis(O=5;E=0.37;R=13.51;P=2.41940019371E-05)

07---------------------mitotic checkpoint(O=3;E=0.16;R=18.75;P=0.000382494856421)

04------------cell division(O=10;E=1.28;R=7.81;P=3.50809359427E-07)

04------------cell organization and biogenesis

05---------------organelle organization and biogenesis(O=15;E=5.52;R=2.72;P=0.000267794721252)

06------------------cytoskeleton organization and biogenesis(O=9;E=2.62;R=3.44;P=0.00103570613076)

07---------------------microtubule-based process(O=9;E=0.93;R=9.68;P=2.01082730727E-07)

08------------------------microtubule cytoskeleton organization and biogenesis(O=7;E=0.41;R=17.07;P=7.99699910868E-08)

09---------------------------spindle organization and biogenesis(O=6;E=0.26;R=23.08;P=7.28798494711E-08)

04------------cellular metabolism

05---------------nucleobase\, nucleoside\, nucleotide and nucleic acid metabolism(O=31;E=18.73;R=1.66;P=0.00113569374385)

06------------------DNA metabolism(O=16;E=4.51;R=3.55;P=5.8588877783E-06)

07---------------------DNA recombination(O=5;E=0.45;R=11.11;P=6.58404021644E-05)

07---------------------DNA repair(O=9;E=1.75;R=5.14;P=4.84233635829E-05)

08------------------------double-strand break repair(O=2;E=0.1;R=20;P=0.00368306003696)

07---------------------DNA replication(O=7;E=1.46;R=4.79;P=0.000538693403181)

07---------------------regulation of DNA metabolism(O=3;E=0.24;R=12.5;P=0.00141969508954)

06------------------regulation of nucleobase\, nucleoside\, nucleotide and nucleic acid metabolism

07---------------------regulation of DNA metabolism(O=3;E=0.24;R=12.5;P=0.00141969508954)

05---------------regulation of cellular metabolism

06------------------regulation of nucleobase\, nucleoside\, nucleotide and nucleic acid metabolism

07---------------------regulation of DNA metabolism(O=3;E=0.24;R=12.5;P=0.00141969508954)

04------------regulation of cellular physiological process(O=30;E=18.04;R=1.66;P=0.0013370862299)

05---------------regulation of cellular metabolism

06------------------regulation of nucleobase\, nucleoside\, nucleotide and nucleic acid metabolism

07---------------------regulation of DNA metabolism(O=3;E=0.24;R=12.5;P=0.00141969508954)

05---------------regulation of progression through cell cycle(O=13;E=3.82;R=3.4;P=7.86562941475E-05)

06------------------cell cycle checkpoint(O=4;E=0.41;R=9.76;P=0.000643149899572)

07---------------------mitotic checkpoint(O=3;E=0.16;R=18.75;P=0.000382494856421)

06------------------regulation of cyclin dependent protein kinase activity(O=3;E=0.45;R=6.67;P=0.00978469831304)

06------------------regulation of mitosis(O=5;E=0.37;R=13.51;P=2.41940019371E-05)

07---------------------mitotic checkpoint(O=3;E=0.16;R=18.75;P=0.000382494856421)

03---------regulation of cellular process(O=30;E=19.09;R=1.57;P=0.00344165081737)

04------------regulation of cellular physiological process(O=30;E=18.04;R=1.66;P=0.0013370862299)

05---------------regulation of cellular metabolism

06------------------regulation of nucleobase\, nucleoside\, nucleotide and nucleic acid metabolism

07---------------------regulation of DNA metabolism(O=3;E=0.24;R=12.5;P=0.00141969508954)

05---------------regulation of progression through cell cycle(O=13;E=3.82;R=3.4;P=7.86562941475E-05)

06------------------cell cycle checkpoint(O=4;E=0.41;R=9.76;P=0.000643149899572)

07---------------------mitotic checkpoint(O=3;E=0.16;R=18.75;P=0.000382494856421)

06------------------regulation of cyclin dependent protein kinase activity(O=3;E=0.45;R=6.67;P=0.00978469831304)

06------------------regulation of mitosis(O=5;E=0.37;R=13.51;P=2.41940019371E-05)

07---------------------mitotic checkpoint(O=3;E=0.16;R=18.75;P=0.000382494856421)

02------physiological process

03---------cellular physiological process(O=67;E=58.7;R=1.14;P=0.00459957343727)

04------------cell cycle(O=23;E=5.4;R=4.26;P=7.3632078069E-10)

05---------------interphase(O=5;E=0.51;R=9.8;P=0.000122920200122)

06------------------interphase of mitotic cell cycle(O=5;E=0.51;R=9.8;P=0.000122920200122)

05---------------M phase(O=14;E=1.52;R=9.21;P=1.14354158563E-10)

06------------------M phase of mitotic cell cycle(O=11;E=1.3;R=8.46;P=3.60193701196E-08)

07---------------------mitosis(O=11;E=1.26;R=8.73;P=2.57254603932E-08)

08------------------------regulation of mitosis(O=5;E=0.37;R=13.51;P=2.41940019371E-05)

09---------------------------mitotic checkpoint(O=3;E=0.16;R=18.75;P=0.000382494856421)

05---------------mitotic cell cycle(O=15;E=1.73;R=8.67;P=5.99335349036E-11)

06------------------interphase of mitotic cell cycle(O=5;E=0.51;R=9.8;P=0.000122920200122)

06------------------M phase of mitotic cell cycle(O=11;E=1.3;R=8.46;P=3.60193701196E-08)

07---------------------mitosis(O=11;E=1.26;R=8.73;P=2.57254603932E-08)

08------------------------regulation of mitosis(O=5;E=0.37;R=13.51;P=2.41940019371E-05)

09---------------------------mitotic checkpoint(O=3;E=0.16;R=18.75;P=0.000382494856421)

05---------------regulation of progression through cell cycle(O=13;E=3.82;R=3.4;P=7.86562941475E-05)

06------------------cell cycle checkpoint(O=4;E=0.41;R=9.76;P=0.000643149899572)

07---------------------mitotic checkpoint(O=3;E=0.16;R=18.75;P=0.000382494856421)

06------------------regulation of cyclin dependent protein kinase activity(O=3;E=0.45;R=6.67;P=0.00978469831304)

06------------------regulation of mitosis(O=5;E=0.37;R=13.51;P=2.41940019371E-05)

07---------------------mitotic checkpoint(O=3;E=0.16;R=18.75;P=0.000382494856421)

04------------cell division(O=10;E=1.28;R=7.81;P=3.50809359427E-07)

04------------cell organization and biogenesis

05---------------organelle organization and biogenesis(O=15;E=5.52;R=2.72;P=0.000267794721252)

06------------------cytoskeleton organization and biogenesis(O=9;E=2.62;R=3.44;P=0.00103570613076)

07---------------------microtubule-based process(O=9;E=0.93;R=9.68;P=2.01082730727E-07)

08------------------------microtubule cytoskeleton organization and biogenesis(O=7;E=0.41;R=17.07;P=7.99699910868E-08)

09---------------------------spindle organization and biogenesis(O=6;E=0.26;R=23.08;P=7.28798494711E-08)

04------------cellular metabolism

05---------------nucleobase\, nucleoside\, nucleotide and nucleic acid metabolism(O=31;E=18.73;R=1.66;P=0.00113569374385)

06------------------DNA metabolism(O=16;E=4.51;R=3.55;P=5.8588877783E-06)

07---------------------DNA recombination(O=5;E=0.45;R=11.11;P=6.58404021644E-05)

07---------------------DNA repair(O=9;E=1.75;R=5.14;P=4.84233635829E-05)

08------------------------double-strand break repair(O=2;E=0.1;R=20;P=0.00368306003696)

07---------------------DNA replication(O=7;E=1.46;R=4.79;P=0.000538693403181)

07---------------------regulation of DNA metabolism(O=3;E=0.24;R=12.5;P=0.00141969508954)

06------------------regulation of nucleobase\, nucleoside\, nucleotide and nucleic acid metabolism

07---------------------regulation of DNA metabolism(O=3;E=0.24;R=12.5;P=0.00141969508954)

05---------------regulation of cellular metabolism

06------------------regulation of nucleobase\, nucleoside\, nucleotide and nucleic acid metabolism

07---------------------regulation of DNA metabolism(O=3;E=0.24;R=12.5;P=0.00141969508954)

04------------regulation of cellular physiological process(O=30;E=18.04;R=1.66;P=0.0013370862299)

05---------------regulation of cellular metabolism

06------------------regulation of nucleobase\, nucleoside\, nucleotide and nucleic acid metabolism

07---------------------regulation of DNA metabolism(O=3;E=0.24;R=12.5;P=0.00141969508954)

05---------------regulation of progression through cell cycle(O=13;E=3.82;R=3.4;P=7.86562941475E-05)

06------------------cell cycle checkpoint(O=4;E=0.41;R=9.76;P=0.000643149899572)

07---------------------mitotic checkpoint(O=3;E=0.16;R=18.75;P=0.000382494856421)

06------------------regulation of cyclin dependent protein kinase activity(O=3;E=0.45;R=6.67;P=0.00978469831304)

06------------------regulation of mitosis(O=5;E=0.37;R=13.51;P=2.41940019371E-05)

07---------------------mitotic checkpoint(O=3;E=0.16;R=18.75;P=0.000382494856421)

03---------metabolism

04------------cellular metabolism

05---------------nucleobase\, nucleoside\, nucleotide and nucleic acid metabolism(O=31;E=18.73;R=1.66;P=0.00113569374385)

06------------------DNA metabolism(O=16;E=4.51;R=3.55;P=5.8588877783E-06)

07---------------------DNA recombination(O=5;E=0.45;R=11.11;P=6.58404021644E-05)

07---------------------DNA repair(O=9;E=1.75;R=5.14;P=4.84233635829E-05)

08------------------------double-strand break repair(O=2;E=0.1;R=20;P=0.00368306003696)

07---------------------DNA replication(O=7;E=1.46;R=4.79;P=0.000538693403181)

07---------------------regulation of DNA metabolism(O=3;E=0.24;R=12.5;P=0.00141969508954)

06------------------regulation of nucleobase\, nucleoside\, nucleotide and nucleic acid metabolism

07---------------------regulation of DNA metabolism(O=3;E=0.24;R=12.5;P=0.00141969508954)

05---------------regulation of cellular metabolism

06------------------regulation of nucleobase\, nucleoside\, nucleotide and nucleic acid metabolism

07---------------------regulation of DNA metabolism(O=3;E=0.24;R=12.5;P=0.00141969508954)

04------------macromolecule metabolism(O=38;E=26.65;R=1.43;P=0.00425371440147)

05---------------biopolymer metabolism(O=33;E=16.74;R=1.97;P=1.7465089088E-05)

06------------------DNA metabolism(O=16;E=4.51;R=3.55;P=5.8588877783E-06)

07---------------------DNA recombination(O=5;E=0.45;R=11.11;P=6.58404021644E-05)

07---------------------DNA repair(O=9;E=1.75;R=5.14;P=4.84233635829E-05)

08------------------------double-strand break repair(O=2;E=0.1;R=20;P=0.00368306003696)

07---------------------DNA replication(O=7;E=1.46;R=4.79;P=0.000538693403181)

07---------------------regulation of DNA metabolism(O=3;E=0.24;R=12.5;P=0.00141969508954)

04------------primary metabolism(O=51;E=40.9;R=1.25;P=0.00954425518841)

05---------------nucleobase\, nucleoside\, nucleotide and nucleic acid metabolism(O=31;E=18.73;R=1.66;P=0.00113569374385)

06------------------DNA metabolism(O=16;E=4.51;R=3.55;P=5.8588877783E-06)

07---------------------DNA recombination(O=5;E=0.45;R=11.11;P=6.58404021644E-05)

07---------------------DNA repair(O=9;E=1.75;R=5.14;P=4.84233635829E-05)

08------------------------double-strand break repair(O=2;E=0.1;R=20;P=0.00368306003696)

07---------------------DNA replication(O=7;E=1.46;R=4.79;P=0.000538693403181)

07---------------------regulation of DNA metabolism(O=3;E=0.24;R=12.5;P=0.00141969508954)

06------------------regulation of nucleobase\, nucleoside\, nucleotide and nucleic acid metabolism

07---------------------regulation of DNA metabolism(O=3;E=0.24;R=12.5;P=0.00141969508954)

04------------regulation of metabolism

05---------------regulation of cellular metabolism

06------------------regulation of nucleobase\, nucleoside\, nucleotide and nucleic acid metabolism

07---------------------regulation of DNA metabolism(O=3;E=0.24;R=12.5;P=0.00141969508954)

03---------regulation of physiological process(O=30;E=18.73;R=1.6;P=0.00252704123742)

04------------regulation of cellular physiological process(O=30;E=18.04;R=1.66;P=0.0013370862299)

05---------------regulation of cellular metabolism

06------------------regulation of nucleobase\, nucleoside\, nucleotide and nucleic acid metabolism

07---------------------regulation of DNA metabolism(O=3;E=0.24;R=12.5;P=0.00141969508954)

05---------------regulation of progression through cell cycle(O=13;E=3.82;R=3.4;P=7.86562941475E-05)

06------------------cell cycle checkpoint(O=4;E=0.41;R=9.76;P=0.000643149899572)

07---------------------mitotic checkpoint(O=3;E=0.16;R=18.75;P=0.000382494856421)

06------------------regulation of cyclin dependent protein kinase activity(O=3;E=0.45;R=6.67;P=0.00978469831304)

06------------------regulation of mitosis(O=5;E=0.37;R=13.51;P=2.41940019371E-05)

07---------------------mitotic checkpoint(O=3;E=0.16;R=18.75;P=0.000382494856421)

04------------regulation of metabolism

05---------------regulation of cellular metabolism

06------------------regulation of nucleobase\, nucleoside\, nucleotide and nucleic acid metabolism

07---------------------regulation of DNA metabolism(O=3;E=0.24;R=12.5;P=0.00141969508954)

02------regulation of biological process(O=30;E=20.43;R=1.47;P=0.00995308119757)

03---------regulation of cellular process(O=30;E=19.09;R=1.57;P=0.00344165081737)

04------------regulation of cellular physiological process(O=30;E=18.04;R=1.66;P=0.0013370862299)

05---------------regulation of cellular metabolism

06------------------regulation of nucleobase\, nucleoside\, nucleotide and nucleic acid metabolism

07---------------------regulation of DNA metabolism(O=3;E=0.24;R=12.5;P=0.00141969508954)

05---------------regulation of progression through cell cycle(O=13;E=3.82;R=3.4;P=7.86562941475E-05)

06------------------cell cycle checkpoint(O=4;E=0.41;R=9.76;P=0.000643149899572)

07---------------------mitotic checkpoint(O=3;E=0.16;R=18.75;P=0.000382494856421)

06------------------regulation of cyclin dependent protein kinase activity(O=3;E=0.45;R=6.67;P=0.00978469831304)

06------------------regulation of mitosis(O=5;E=0.37;R=13.51;P=2.41940019371E-05)

07---------------------mitotic checkpoint(O=3;E=0.16;R=18.75;P=0.000382494856421)

03---------regulation of enzyme activity

04------------regulation of transferase activity

05---------------regulation of protein kinase activity

06------------------regulation of cyclin dependent protein kinase activity(O=3;E=0.45;R=6.67;P=0.00978469831304)

03---------regulation of physiological process(O=30;E=18.73;R=1.6;P=0.00252704123742)

04------------regulation of cellular physiological process(O=30;E=18.04;R=1.66;P=0.0013370862299)

05---------------regulation of cellular metabolism

06------------------regulation of nucleobase\, nucleoside\, nucleotide and nucleic acid metabolism

07---------------------regulation of DNA metabolism(O=3;E=0.24;R=12.5;P=0.00141969508954)

05---------------regulation of progression through cell cycle(O=13;E=3.82;R=3.4;P=7.86562941475E-05)

06------------------cell cycle checkpoint(O=4;E=0.41;R=9.76;P=0.000643149899572)

07---------------------mitotic checkpoint(O=3;E=0.16;R=18.75;P=0.000382494856421)

06------------------regulation of cyclin dependent protein kinase activity(O=3;E=0.45;R=6.67;P=0.00978469831304)

06------------------regulation of mitosis(O=5;E=0.37;R=13.51;P=2.41940019371E-05)

07---------------------mitotic checkpoint(O=3;E=0.16;R=18.75;P=0.000382494856421)

04------------regulation of metabolism

05---------------regulation of cellular metabolism

06------------------regulation of nucleobase\, nucleoside\, nucleotide and nucleic acid metabolism

07---------------------regulation of DNA metabolism(O=3;E=0.24;R=12.5;P=0.00141969508954)

02------response to stimulus

03---------response to abiotic stimulus

04------------response to chemical stimulus

05---------------response to drug(O=2;E=0.12;R=16.67;P=0.00545431475713)

03---------response to endogenous stimulus(O=9;E=1.97;R=4.57;P=0.000121844627465)

04------------response to DNA damage stimulus(O=9;E=1.93;R=4.66;P=0.000104029149443)

05---------------DNA repair(O=9;E=1.75;R=5.14;P=4.84233635829E-05)

06------------------double-strand break repair(O=2;E=0.1;R=20;P=0.00368306003696)

03---------response to stress

04------------response to DNA damage stimulus(O=9;E=1.93;R=4.66;P=0.000104029149443)

05---------------DNA repair(O=9;E=1.75;R=5.14;P=4.84233635829E-05)

06------------------double-strand break repair(O=2;E=0.1;R=20;P=0.00368306003696)

01---molecular\_function

02------binding

03---------nucleic acid binding(O=26;E=16.76;R=1.55;P=0.0094704406456)

04------------DNA binding

05---------------damaged DNA binding(O=3;E=0.35;R=8.57;P=0.00447763502957)

03---------nucleotide binding(O=21;E=11.9;R=1.76;P=0.00503999582956)

04------------purine nucleotide binding(O=19;E=10.21;R=1.86;P=0.00444520983836)

05---------------adenyl nucleotide binding(O=18;E=8.4;R=2.14;P=0.00118547416682)

06------------------ATP binding(O=18;E=8.11;R=2.22;P=0.000787576208882)

02------transporter activity

03---------ion transporter activity

04------------anion transporter activity

05---------------organic anion transporter activity(O=2;E=0.06;R=33.33;P=0.00120866771215)

01---cellular\_component

02------cell

03---------intracellular(O=62;E=46.8;R=1.32;P=1.48938455711E-05)

04------------chromosome(O=7;E=1.87;R=3.74;P=0.00232225082456)

05---------------chromosome\, pericentric region(O=3;E=0.2;R=15;P=0.000867024448873)

06------------------kinetochore(O=3;E=0.18;R=16.67;P=0.000615855537189)

04------------cytoskeleton

05---------------microtubule cytoskeleton(O=9;E=1.54;R=5.84;P=1.65805599357E-05)

06------------------microtubule(O=6;E=1.06;R=5.66;P=0.00054723812892)

06------------------spindle(O=4;E=0.35;R=11.43;P=0.000303045626565)

04------------intracellular organelle(O=55;E=38.86;R=1.42;P=3.41369602151E-05)

05---------------intracellular membrane-bound organelle(O=51;E=33.59;R=1.52;P=1.45411694448E-05)

06------------------nucleus(O=46;E=21.55;R=2.13;P=1.05057832233E-09)

07---------------------nuclear membrane(O=4;E=0.75;R=5.33;P=0.00619543706665)

07---------------------nucleoplasm

08------------------------transcription factor complex

09---------------------------histone acetyltransferase complex(O=2;E=0.08;R=25;P=0.00237243551213)

10------------------------------TIP60 histone acetyltransferase complex(O=2;E=0.08;R=25;P=0.00237243551213)

05---------------intracellular non-membrane-bound organelle(O=19;E=9.13;R=2.08;P=0.00109879892467)

06------------------chromosome(O=7;E=1.87;R=3.74;P=0.00232225082456)

07---------------------chromosome\, pericentric region(O=3;E=0.2;R=15;P=0.000867024448873)

08------------------------kinetochore(O=3;E=0.18;R=16.67;P=0.000615855537189)

06------------------cytoskeleton

07---------------------microtubule cytoskeleton(O=9;E=1.54;R=5.84;P=1.65805599357E-05)

08------------------------microtubule(O=6;E=1.06;R=5.66;P=0.00054723812892)

08------------------------spindle(O=4;E=0.35;R=11.43;P=0.000303045626565)

06------------------kinetochore(O=3;E=0.18;R=16.67;P=0.000615855537189)

06------------------spindle(O=4;E=0.35;R=11.43;P=0.000303045626565)

04------------nucleus(O=46;E=21.55;R=2.13;P=1.05057832233E-09)

05---------------nuclear membrane(O=4;E=0.75;R=5.33;P=0.00619543706665)

05---------------nucleoplasm

06------------------transcription factor complex

07---------------------histone acetyltransferase complex(O=2;E=0.08;R=25;P=0.00237243551213)

08------------------------TIP60 histone acetyltransferase complex(O=2;E=0.08;R=25;P=0.00237243551213)

03---------membrane

04------------endomembrane system

05---------------nuclear membrane(O=4;E=0.75;R=5.33;P=0.00619543706665)

04------------organelle membrane

05---------------nuclear membrane(O=4;E=0.75;R=5.33;P=0.00619543706665)

02------organelle(O=55;E=38.86;R=1.42;P=3.41369602151E-05)

03---------intracellular organelle(O=55;E=38.86;R=1.42;P=3.41369602151E-05)

04------------intracellular membrane-bound organelle(O=51;E=33.59;R=1.52;P=1.45411694448E-05)

05---------------nucleus(O=46;E=21.55;R=2.13;P=1.05057832233E-09)

06------------------nuclear membrane(O=4;E=0.75;R=5.33;P=0.00619543706665)

06------------------nucleoplasm

07---------------------transcription factor complex

08------------------------histone acetyltransferase complex(O=2;E=0.08;R=25;P=0.00237243551213)

09---------------------------TIP60 histone acetyltransferase complex(O=2;E=0.08;R=25;P=0.00237243551213)

04------------intracellular non-membrane-bound organelle(O=19;E=9.13;R=2.08;P=0.00109879892467)

05---------------chromosome(O=7;E=1.87;R=3.74;P=0.00232225082456)

06------------------chromosome\, pericentric region(O=3;E=0.2;R=15;P=0.000867024448873)

07---------------------kinetochore(O=3;E=0.18;R=16.67;P=0.000615855537189)

05---------------cytoskeleton

06------------------microtubule cytoskeleton(O=9;E=1.54;R=5.84;P=1.65805599357E-05)

07---------------------microtubule(O=6;E=1.06;R=5.66;P=0.00054723812892)

07---------------------spindle(O=4;E=0.35;R=11.43;P=0.000303045626565)

05---------------kinetochore(O=3;E=0.18;R=16.67;P=0.000615855537189)

05---------------spindle(O=4;E=0.35;R=11.43;P=0.000303045626565)

03---------membrane-bound organelle(O=51;E=33.59;R=1.52;P=1.45411694448E-05)

04------------intracellular membrane-bound organelle(O=51;E=33.59;R=1.52;P=1.45411694448E-05)

05---------------nucleus(O=46;E=21.55;R=2.13;P=1.05057832233E-09)

06------------------nuclear membrane(O=4;E=0.75;R=5.33;P=0.00619543706665)

06------------------nucleoplasm

07---------------------transcription factor complex

08------------------------histone acetyltransferase complex(O=2;E=0.08;R=25;P=0.00237243551213)

09---------------------------TIP60 histone acetyltransferase complex(O=2;E=0.08;R=25;P=0.00237243551213)

04------------organelle membrane

05---------------nuclear membrane(O=4;E=0.75;R=5.33;P=0.00619543706665)

03---------non-membrane-bound organelle(O=19;E=9.13;R=2.08;P=0.00109879892467)

04------------intracellular non-membrane-bound organelle(O=19;E=9.13;R=2.08;P=0.00109879892467)

05---------------chromosome(O=7;E=1.87;R=3.74;P=0.00232225082456)

06------------------chromosome\, pericentric region(O=3;E=0.2;R=15;P=0.000867024448873)

07---------------------kinetochore(O=3;E=0.18;R=16.67;P=0.000615855537189)

05---------------cytoskeleton

06------------------microtubule cytoskeleton(O=9;E=1.54;R=5.84;P=1.65805599357E-05)

07---------------------microtubule(O=6;E=1.06;R=5.66;P=0.00054723812892)

07---------------------spindle(O=4;E=0.35;R=11.43;P=0.000303045626565)

05---------------kinetochore(O=3;E=0.18;R=16.67;P=0.000615855537189)

05---------------spindle(O=4;E=0.35;R=11.43;P=0.000303045626565)

02------protein complex

03---------transcription factor complex

04------------histone acetyltransferase complex(O=2;E=0.08;R=25;P=0.00237243551213)

05---------------TIP60 histone acetyltransferase complex(O=2;E=0.08;R=25;P=0.00237243551213)
